# Users Of Veteran-Directed Care And Other Purchased Care Have Similar Hospital Use And Costs Over Time

**DOI:** 10.1377/hlthaff.2019.00020

**Published:** 2019-06

**Authors:** Yingzhe Yuan, Kali S. Thomas, Austin B. Frakt, Steven D. Pizer, Melissa M. Garrido

**Affiliations:** Partnered Evidence-Based Policy Resource Center at the Veterans Affairs (VA) Boston Healthcare System; Boston University School of Public Health, both in Massachusetts.; Center of Innovation in Long-Term Services and Supports at the Providence VA Medical Center; Center for Gerontology and Healthcare Research,Brown University School of Public Health, all in Providence, Rhode Island.; Partnered Evidence-Based Policy Resource Center at the VA Boston Healthcare System; Boston University School of Public Health; Harvard T. H. Chan School of Public Health, all in Boston.; Partnered Evidence-Based Policy Resource Center at the VA Boston Healthcare System; health law, policy, and management, Boston University School of Public Health.; Partnered Evidence-Based Policy Resource Center at the VA Boston Healthcare System; health law, policy, and management, Boston University School of Public Health.

## Abstract

The Veteran-Directed Care (VDC) program facilitates independent community living among adults with multiple chronic conditions and functional limitations. Family caregivers value the choice and flexibility afforded by VDC, but rigorous evidence to support its impact on health care costs and use is needed. We identified veterans enrolled in VDC in fiscal year 2017 and investigated differences in hospital admissions and costs after initial receipt of VDC services. We compared VDC service recipients to a matched comparison group of veterans receiving homemaker or home health aide, home respite, and adult day health care services and found similar decreases in hospital use and costs from before to after enrollment in the groups. Further investigation into trends of nursing home use, identification of veterans most likely to benefit from VDC, and relative costs of operating VDC versus other purchased care programs is needed, but our results suggest that VDC remains a valuable option for supporting veterans and caregivers.

Unpaid family caregivers make up a large portion of the workforce caring for adults with multiple chronic conditions.^1,2^ Caregiver burden is common and associated with adverse consequences for care recipients.^3–5^ Supporting caregivers is of special concern to the Veterans Health Administration (VHA) (as made clear in, for example, the Care-givers and Veterans Omnibus Health Services Act of 2010) and a critical component of its Choose Home Initiative to provide long-term care in the community where appropriate and desired.^6^

One way the VHA facilitates independence and supports caregivers is through the Veteran-Directed Care (VDC) program of the VHA’s Office of Geriatrics and Extended Care Services. The VDC program is participant directed and modeled after the Medicaid Cash and Counseling Demonstration and Evaluation, which was associated with improved care satisfaction, fewer unmet needs for assistance with activities of daily living, and reduced caregiver physical and emotional strain.^7–10^ Although referral criteria are flexible, the target recipients are veterans who have at least three limitations in activities of daily living, have cognitive limitations, require at least twenty hours per week of home care, or are otherwise eligible for nursing home level care. Veterans receive monthly budgets and purchase services or goods in consultation with options counselors at aging and disability network agencies. VDC provides flexibility to enroll-ees and enables them to hire family members, friends, or neighbors as paid caregivers. VDC also provides support to caregivers, including caregiver training and burden monitoring.^11^

Interviews with VDC coordinators at Veterans Affairs medical centers and aging and disability network agencies suggest that VDC is meeting veterans’ needs and enabling them to remain safely in the community.^12,13^ VDC participants call the program “life changing” and unanimous ly recommend it to other veterans.^14^ Outside the VHA, enrollees in participant-directed programs are more satisfied than recipients of traditional agency-based care.^8,15^

Family caregivers value the choice and flexibility afforded by participant-directed programs.^16^

They reportreduced stress associated with using paid caregivers through VDC, because of the control they have over hiring decisions and the ability to work with a consistent and reliable paid caregiver over an extended timeframe.^17^ Family caregivers in VDC value scheduling care-giving help when it is needed, rather than according to an agency’s schedule.^17^ Improved caregiver satisfaction and emotional well-being have been reported in other participant-directed programs.^16,18^

AARP recently highlighted VDC as a promising practice for facilitating independence in the community.^11^ VDC allows veterans and care-givers to purchase goods and services that best meet veterans’ needs. This autonomy, flexibility, and choice may increase satisfaction with care and reduce caregiver stress and burden. Given the link between emotional or illness burden among caregivers and unnecessary health care use among care recipients,^4,5,18,19^ VDC likely promotes reductions in veterans’ unnecessary service use and increased ability to remain in the community.^20,21^ To the extent that VDC helps prevent unnecessary hospitalizations, it may also decrease health care costs among people with chronic conditions and functional limitations.^22^ These people are among the patient populations with the highest health care costs.^22^ However, the relationship between VDC and hospitalization rates and costs is unknown.

VDC evaluations to date have been limited by the lack of a control group or systematic data on health care use or costs. Outside the VHA, evaluations of participant-directed programs’ impact on health care use and costs have been limited to analyses of people who volunteered to be randomly assigned to one of these programs or to usual care (who may systematically differ from those unwilling to be randomly assigned) or of samples in which unobserved differences between participants and nonparticipants may have influenced results.^23,24^

In this study we evaluated the incidence and frequency of VHA hospital admissions, ambulatory care–sensitive admissions, and costs associated with inpatient care in the year after the initial receipt of a VDC service. We compared outcomes among VDC enrollees and people receiving other VHA purchased care services (homemaker or home health aide, home respite, or contract adult day health care) that are targeted to veterans with serious functional or cognitive impairment. Of these services, VDC is unique in the degree of choice that it affords veterans and their family caregivers. Because of its positive reception among caregivers, we hypothesized that VDC might be associated with reduced preventable hospitalizations over time, compared to other purchased care services.

Given VDC’s popularity, there is interest in expanding the program.^25^ Expansion would be further justified if health care outcomes for veterans enrolled in VDC were at least noninferior to outcomes for veterans enrolled in other purchased care programs. Our results will inform both the VHA and other organizations that seek to allocate resources to interventions most likely to improve care for seriously ill people and their family caregivers.

## Study Data And Methods

### Sample

Our sample included 37,407 veterans who received at least one VHA purchased care service (Veteran-Directed Care or homemaker or home health aide, home respite, or contract adult day health care) in fiscal year 2017 in one of thirty-eight Department of Veterans Affairs (VA) medical centers. Twenty-one of these medical centers had active VDC programs in FY 2017, defined as having at least five patients per month who could be identified as receiving VDC services for at least seven months of the year. Forty-four other medical centers had VDC programs but were excluded because they had low enrollment or did not distinguish between VDC and other purchased care when recording service use. The other seventeen sites in our sample had indicated interest in implementing VDC but had not yet done so by FY 2017.^25^ We limited inactive comparison sites to those that had indicated interest in offering VDC and were thus likely to be similar to active sites in engagement in delivering alternate care models.

All veterans in our sample had at least one inpatient or outpatient VHA visit in the year before receipt of a purchased care service. The VDC group included veterans who had their first VDC visit recorded in FY 2017 and who did not have a previous record of receiving a VDC service (*n* = 965). VDC patients were included regard less of their use of other purchased care services. We created two comparison groups to control for different sources of confounding in our analyses. The first included veterans who received a purchased care service other than a VDC service at any of the sites with an active VDC program in FY 2017—the comparison group at active sites (*n* = 21,117). This group was included to accountfor site differences in care patterns. The second included veterans who received a purchased care service, but not a VDC service, at any of the seventeen sites in our sample without an active VDC program—the comparison group at inactive sites that were interested in offering VDC (*n* = 15,325). This group was included to controlfor individual-level selection bias within sites (where factors such as functional limitations may be simultaneously associated with VDC use and outcomes).

We examined VHA hospitalizations and costs in the year before and the year after the index date. For patients in the intervention group, the first VDC service date was the index date. For comparison-group patients, the first service date of a VHA purchased care service in FY 2017 was the index date. Our primary analyses included all patients with any use of a qualifying purchased care service, regardless of service duration. Patients were followed for 360 days after the index date or until death, whichever came first. Of our sample, 21.9 percent died before the end of the follow-up period, with similar mortality rates across treatment and comparison groups (exhibit 1). As a result, our data set included 848,516 person-month observations.

This study was approved by the Boston VA Healthcare System Institutional Review Board.

### Data And Measures

Data on inpatient and outpatient care, purchased care, vital statistics, mortality risk, risk-adjustment scores, and health care costs came from the VA’s Corporate Data Warehouse, Managerial Cost Accounting National Data Extracts, Health Economics Resource Center Discharge data set, and the weekly Care Assessment Needs (CAN) score report.

Our primary outcome was monthly incidence of an acute care admission at a VA medical center. In addition, we calculated hospitalization costs per month for each patient (see the online appendix).^26^ Twenty-seven patients were still in the hospital at the end of our 360-day follow-up period and had missing cost data.We also calculated incidence and costs associated with ambulatory care–sensitive hospitalizations, using the Prevention Quality Indicators of the Agency for Healthcare Research and Quality.^27^

To characterize recipients of VDC and other purchased care services, we measured age at index date, sex, race, Medicaid eligibility, urban or rural patient location, and service-connected disability. We assessed receipt of Veterans Benefits Administration aid and attendance benefits, which are provided to veterans with functional limitations. We measured one-year mortality probability with the CAN one-year mortality score nearest to the index date (range 0–99, where 99 indicates highest probability of death).^28^ To measure chronic disease burden, we included the number of Elixhauser comorbidities over the year before the index date and the latest Nosos risk score before the index date (where scores indicate the degree to which a patient’s next-year health care costs are predicted to differ from the VA national average [1 = national average]).^29,30^ We also measuredwhether veterans had diagnoses associated with greater care needs: spinal cord injury, traumatic brain injury, and dementia. We described mortality over the follow-up period and duration of purchased care service use after the index date.

### Analytic Strategy

We described veterans enrolled in VDC in FY 2017 and investigated differences in the incidence of any and ambulatory care–sensitive hospital admissions and hospitalization costs after initial VDC service receipt. We compared characteristics of veterans in our intervention and comparison groups using analysis-of-variance tests, chi-square tests, Kruskal-Wallis tests, and standardized differences.

Differences in outcomes from before to after first VDC use were compared to differences in outcomes from before to after first use of other purchased care programs in FY 2017 in both comparison groups.

Our primary analyses were fixed effects models that included indicators for enrollment in VDC after the index date, enrollment in other purchased care services at sites where VDC was not available, and time (in months). The models implicitly controlled for time-invariant site and patient characteristics. We used logistic models to explore the relationship between VDC and hospital admissions. Because fixed effects logistic models can be biased away from the null for panel data, we reestimated these models with population-averaged models, which are biased toward the null.^31^ We used linear fixed effects models to explore relationships between VDC and inpatient costs among all veterans in the sample. To better approximate the cost data distribution, we ran population-averaged panel-data models with gamma distribution and log link (see the appendix).^26^

To improve precision, we reran analyses on a matched cohort of patients who had similar health care needs and sociodemographic characteristics but who differed on whether they had used VDC.We performed coarsened exact matching across all sites in our sample and within sites that offered VDC.

We also explored the robustness of our results to the definition of the follow-up period, sample selection criteria, and service use duration (see the appendix).^26^

### Limitations

Our study had several limitations. First, we did not analyze care received outside of the VHA (for example, care paid for by Medicare or Medicaid).^32,33^ In addition, thenumber of veterans enrolled in VDC is relatively small, which limited our ability to detect significant associations between VDC participation and our outcomes. However, a strength of this study is that we used the most current VHA data available. Waiting for Medicare and Medicaid data would have delayed the results for several years.

Second, for both intervention patients and control patients selected from sites with active VDC programs, there is a possibility that a VDC service was received but coded as homemaker or home health aide before FY 2017. The stop code for VDC was not introduced until FY 2017, and code implementation varied by site. This potential measurement error could have biased our results toward the null.

Third, there could have been unmeasured changes in functional limitations, care preferences, or other factors that are simultaneously associated with VDC service receipt, and changes in health care use and costs. However, our analyses allowed us to explore changes in hospitalizations and costs and to isolate them from the impact of important time-invariant confounders, including functional limitations and chronic disease burden.

## Study Results

### Descriptive Results

Veterans who received a Veteran-Directed Care service were younger than veterans who received other purchased care services in active and inactive sites (mean ages: 74, 77, and 76, respectively) (exhibit 1). However, VDC veterans had higher mean Nosos scores(3.8), compared to veterans in the active or inactive comparison group (3.0 and 3.3, respectively). VDC enrollees were more likely to have a spinal cord injury, to have received aid and attendance benefits, and to reside in a rural location than were veterans in either comparison group. They were also more likely to have received at least one service for each month of the follow-up period (see the appendix).^26^

In the twelfth month before receiving services, more VDC recipients (5.0 percent) were hospitalized than patients in the active or inactive comparison groups (3.3 percent and 3.7 percent, respectively) (see exhibit 2 and the appendix).^26^In the sixth month before receiving services, this pattern persisted. However, in the sixth month after receiving services, hospitalization incidence was similar across groups. This pattern persisted in the twelfth month after receiving services: 4.7 percent of VDC recipients, 4.2 percent of patients in the active comparison group, and 5.0 percent of patients in the inactive comparison group were hospitalized (exhibit 2).

In the twelfth month before the index date, hospitalized VDC patients had higher mean monthly hospital costs ($1,624) than hospitalized patients in the active and inactive comparison groups ($752 and $749, respectively) (see exhibit 3 and the appendix).^26^ During the sixth month before receiving services, mean monthly hospital costs were $2,131 for VDC patients, $1,054 for patients in the active comparison group, and $974 for patients in the inactive comparison group. In the sixth month after receiving services, mean monthly costs were $1,569, $1,170, and $1,049, respectively. In the twelfth month after receiving services, VDC patients still had higher mean monthly hospital costs ($1,331)than patients in the active or inactive comparison group ($1,007 and $1,027, respectively), but the difference between mean hospital costs among VDC patients and comparison-group patients had decreased.

Ambulatory care–sensitive hospitalizations were relatively infrequent for each group at each time point (0.5–1.0 percent) (exhibit 2). As a result, data on the costs of such hospitalizations were available for only three to fourteen veterans per month in the VDC group, which precluded further analyses on the costs among our sample.

### All Hospital Admissions

In our logistic fixed effects model, VDC service receipt was not significantly associated with changes in hospital admissions over time, relative to receipt of other purchased care services (odds ratio: 0.83) (exhibit 4).

When we matched the entire sample on socio-demographic and clinical characteristics and ran a fixed effects model, the relationship between VDC service receipt and a decrease in hospital admissions over time had a similar magnitude but became significant (defined as p < 0:05) (exhibit 4). We no longer observed a significant relationship when we matched the sample among recipients in sites with active VDC programs (full results of all models are in the appendix).^26^

When we ran population-averaged models in our unmatched and matched samples, we did not observe a significant relationship between VDC service receipt and changes in hospital admissions over time.

To account for the possibility that VDC and other purchased care services may take time to affect outcomes, we explored how the results of our fixed effects model changed when we redefined the post period to begin two months after the index date. Changes in hospital admissions across groups were not significant.

### Ambulatory Care–Sensitive Hospitalizations

In our fixed effects model, the association between VDC service receipt and ambulatory care–sensitive hospitalizations was not significant in our unmatched (OR: 1.09) or matched (OR: 1.15 [across all sites]) samples (see exhibit 4 and the appendix).^26^ We observed similar results when matching the sample among recipients in sites with active VDC programs, across different model specifications, and with different definitions of the post period.

### All-Cause Hospitalization Costs

In our fixed effects model, VDC service receipt was significantly associated with changes in hospital costs over time, relative to receipt of other purchased care services (average incremental effect: −$358) (exhibit 4). However, when we ran population-averaged models that better accounted for the skewed nature of the cost data, the relationship was no longer significant in our unmatched sample (average incremental effect: −$274).

Although VDC service receipt was significantly associated with changes in hospital costs in fixed effects models in matched samples, it was not robust to model specification. VDC service re ceipt was associated with reduced hospital costs in only one matched population–averaged model (the matched entire sample).

When we redefined the post period to begin two months after the index date, changes in hospital costs across groups were no longer significant (see the appendix).^26^

### Robustness To Sample Selection Criteria And Service Use Duration

Our primary analysis included veterans who did and did not have a history of receiving purchased care services in the year before first VDC service receipt. However, VDC is often used in a stepped care model, and it is sometimes provided when a clinician determines that a veteran needs more care than is practical to provide through other purchased care service programs. For this reason, we reran our models with the treatment group restricted to the 511 veterans who received at least one purchased care service in the year before the VDC index date. There was no significant association among VDC service receipt and either all-cause costs or all-cause or ambulatory care sensitive admissions in this subset.

When we explored robustness to minimum purchased care service use duration (three or six months), there was no evidence of an association among VDC service receipt and all-cause or ambulatory care–sensitive admissions (see the appendix).^26^ Reduced costs were observed in fixed effects models, but these results were not robust to model specification.

Among nondecedents, VDC service receipt was associated with 15–19 percent lower odds of all-cause hospital admissions and with lower hospitalization costs (average incremental effect: −$213; 95% confidence interval: −$373, −$53) in our matched samples (see the appendix).^26^ No associations with ambulatory care sensitive hospitalizations was observed among nondecedents.

## Discussion

Using the most comprehensive data available to date on hospital use and costs for recipients of Veteran-Directed Care services, we found similar decreases in all-cause hospital use and costs from before to after enrollment in VDC and receipt of other purchased care services. This occurred despite VDC enrollees’ being more medically complex: Compared to other enrollees, VDC enrollees were more likely to receive aid and attendance benefits and to have a spinal cord injury, and they had higher Nosos scores. In addition, VDC has been received positively by veterans and their caregivers.^14,17^ Further investigation into trends of other outcomes—including nursing home use, identification of veterans most likely to benefit from VDC, and the relative costs of operating VDC versus other purchased care programs—remains to be completed. Our hypotheses that VDC would be associated with reductions in hospitalizations and costs over time, relative to purchased care services, were supported only in selected subsamples and were not robust to the definition of the postintervention period. Ambulatory care–sensitive hospitalizations remained relatively infrequent over the study period for all VDC and comparison-group patients.

VDC was initiated in 2009 but is not yet operational nationwide. Policy makers and VHA leaders need timely evidence to guide decision making about expansion. A recent Cochrane review called for more evidence on health care use and economic analyses focused on home-based care programs.^34^ This study adds to the existing literature by exploring patterns of hospital costs and use in a sample facing real-world decisions about enrollment in participant-directed programs. Moreover, our comparison groups allowed us to control for within- and across-site differences in care for chronically ill people. Our results demonstrate similar trajectories of outcomes after receipt of a variety of purchased care services.

Future evaluations should examine postacute care use and trajectories of care for longer than a twelve-month period to understand potential differences in outcomes after patients’ needs stabilize. Given that veterans may seek services outside of the VHA,^32,33^ future evaluations should examine the effects of VDC on Medicare and Medicaid use and costs. In addition, future work should evaluate VDC’s impact on veterans’ care-givers, compared to the impact of other purchased care services.

These early results will directly inform VHA policy makers’ efforts to identify optimal bundles of long-term services and supports for the growing number of veterans who are entitled to receive these services through the VHA. VDC is noninferior to other purchased care services in terms of hospital use and cost trajectories, despite being targeted to veterans with greater care needs. VDC allows a degree of flexibility and personal choice in arranging long-term services and supports that is not available in other programs. This flexibility is highly valued by family caregivers and veterans^14,17^ and facilitates the receipt of long-term services and supports among veterans living in rural areas. Although paying for programs with “positive social valence” is desired,^35^ a budget impact analysis of the VDC remains to be conducted. Shifting budgetary sources for VDC over recent years and the lack of a means for accurate cost accounting for the program at the national level preclude a national budget impact analysis at this time.

These results also may serve as a useful benchmark for future evaluations of the VA’s Program of Comprehensive Assistance for Family Care-givers (PCAFC), which provides training and a stipend to family caregivers of veterans injured in the line of duty on or after September 11, 2001. Eligibility for caregivers of veterans injured in the line of duty at any time was granted through the VA Maintaining Internal Systems and Strengthening Integrated Outside Networks (MISSION) Act of 2018. However, this program does not include options counseling, and the stipends are often lower in the PCAFC than in VDC.^36,37^ In a comparison of PCAFC enrollees and nonenrollees, Courtney Van Houtven and colleagues found similar acute care use among the two groups, but higher costs and outpatient use among enrollees than nonenrollees.^38^ A direct comparison of the PCAFC and VDC may help determine whether training or options counseling in addition to financial support for families of people with serious illness is more useful in reducing hospitalizations and associated costs. A comparison of caregiver satisfaction in the PCAFC and VDC is also under way.^39^

## Conclusion

Veterans enrolled in Veteran-Directed Care had indicators of higher chronic disease burden and more functional limitations than veterans enrolled in other purchased care service programs but experienced similar decreases in hospital use and costs from before to after enrolling in services. Given VDC’s popularity among veterans and caregivers, it is a valuable model for supporting medically complex patients with multiple chronic conditions and functional or cognitive limitations who are living in the community. To allocate resources more efficiently, future work should identify the optimal target population for VDC and the optimal methods for using the stipend to facilitate independent community living and support caregivers. ▪

## Supplementary Material

1

## Figures and Tables

**EXHIBIT 2:**
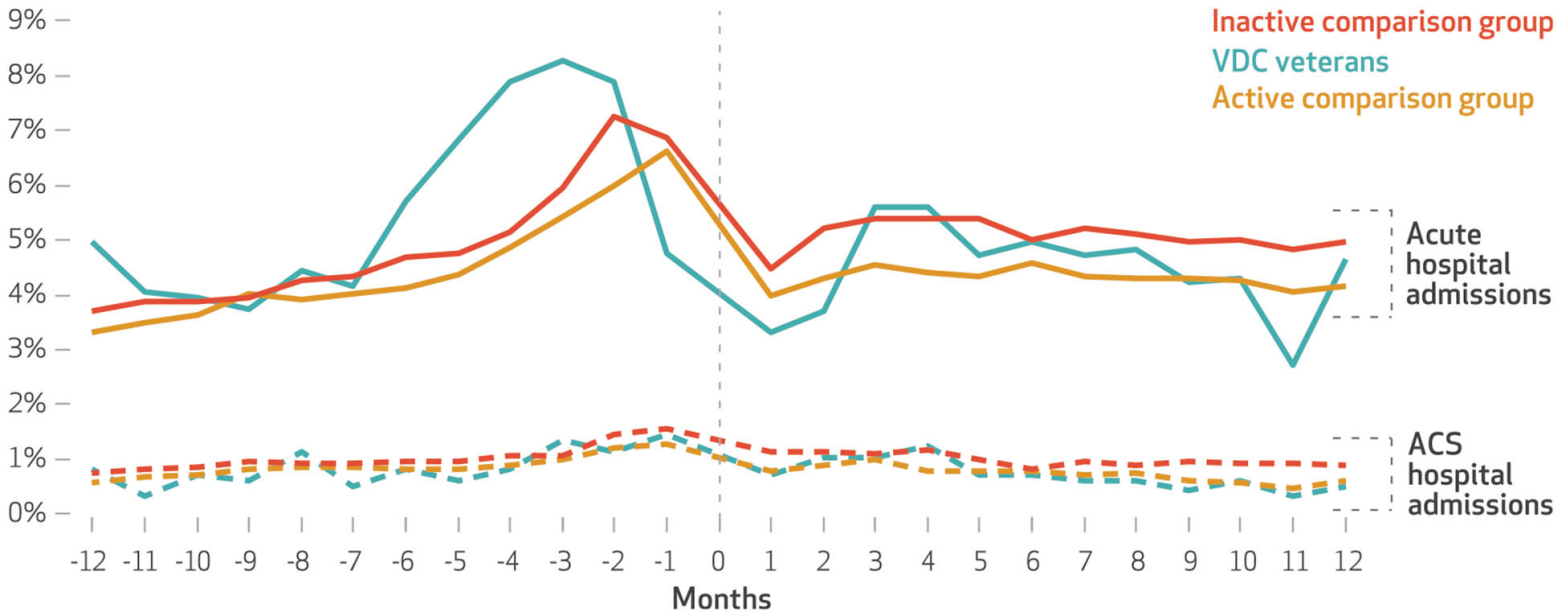
Percent of veterans with acute and ambulatory care–sensitive (ACS) hospital admissions, by month before and after the start of service receipt **SOURCE** Authors’ analysis of aggregated encounter data for 2015–18 from the Veterans Health Administration. **NOTES** The months are thirty-day periods relative to the service initiation date of the Veteran-Directed Care (VDC) program or another purchased care program. Only hospitalizations from the Veterans Health Administration were included. VDC veterans and the “active comparison group” and “inactive comparison group” are explained in the notes to exhibit 1.

**EXHIBIT 3:**
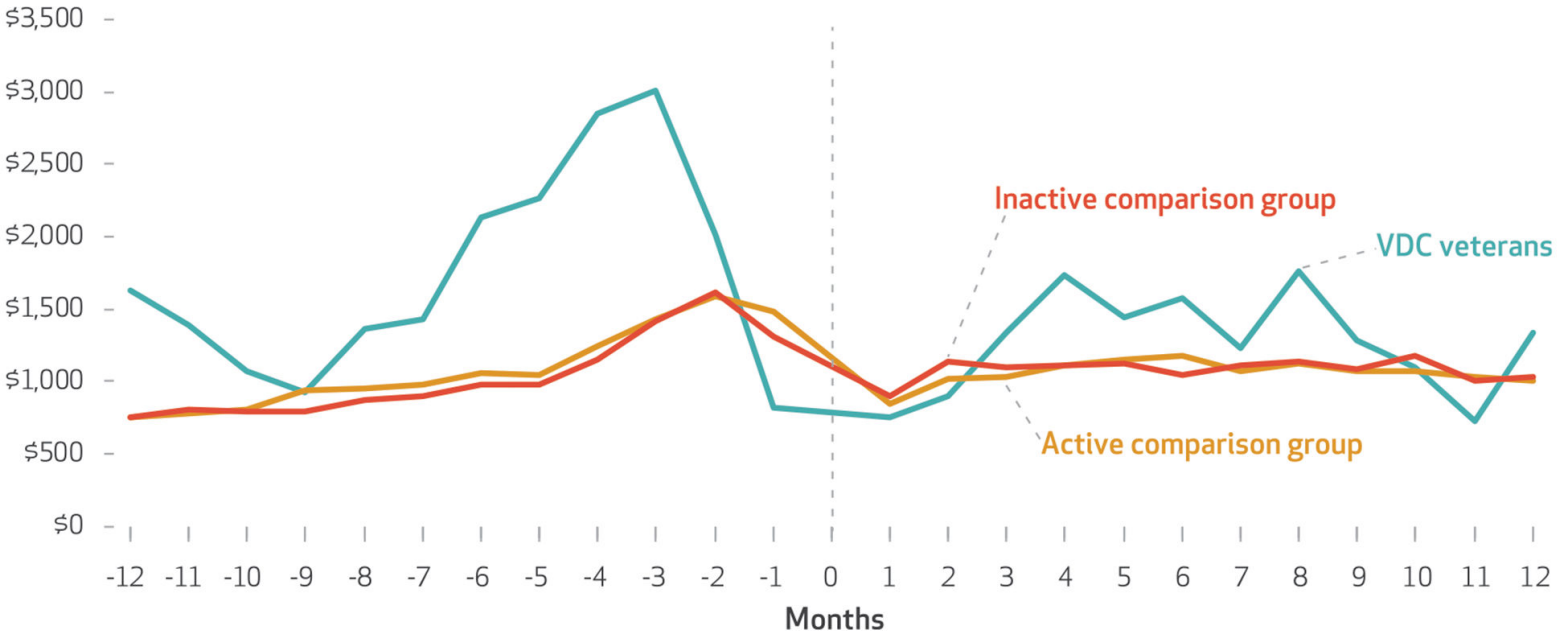
Mean cost of veterans’ inpatient care, by month before and after the start of service receipt **SOURCE** Authors’ analysis of aggregated encounter data and all-cause hospitalization cost data for 2015–18 from the Veterans Health Administration. NOTES Veteran-Directed Care (VDC) veterans and the “active comparison group” and “inactive comparison group” are explained in the notes to exhibit 1. The months are explained in the notes to exhibit 2. Costs include only those attributed to an acute hospitalization in the Veterans Health Administration. Two hospitalizations were excluded as extreme outliers. Cost data for twenty-seven hospitalizations that lasted after the end of the study were excluded.

**EXHIBIT 1:** Characteristics of enrollees in the Veteran-Directed Care (VDC) program and comparison groups **SOURCE** Authors’ analysis of aggregated patient data for 2015–18 from the Veterans Health Administration. **NOTES** VDC veterans were those at a site with an active VDC program who received their first VDC visit in fiscal year 2017. “Active comparison group” refers to veterans who received a purchased care service (homemaker or home health aide, home respite, or contract adult day health care), but not a VDC service, at a site with an active VDC program (defined in the text) in FY 2017. “Inactive comparison group” refers to veterans who received a purchased care service, but not a VDC service, at any of the seventeen sites in our sample without an active VDC program in FY 2017. There were missing observations for some characteristics: 12 for urban location, 3 for enrollment priority, 6,698 for Medicaid eligibility, 12 for receipt of aid and attendance benefits (explained in the text), 1 for age, 835 for the mean Nosos risk score (explained in the text), and 373 for the mean Care Assessment Needs (CAN) score (explained in the text). We used chi-square tests to compare the differences across the three groups in sex; urban location; enrollment priority; Medicaid eligibility; receipt of aid and attendance benefits; and traumatic brain injury, dementia, and spinal cord injury admissions. Analysis-of-variance tests were run to compare differences across the three groups in age, Elixhauser Comorbidity Index score, Nosos risk score, and CAN score. SD is standard deviation.

	Comparison group				
Characteristic	Inactive (n = 15,325)	Active (n = 21,117)	VDC veterans (n = 965)	All (N = 37,407)	p value
Male (%)	94.3	94.3	94.6	94.3	
White (%)	76.5	74.5	72.1	75.2	****
Urban location (%)	60.6	65.8	58.7	63.5	***
Enrollment priority (%)	15.0	14.4	19.7	14.8	****
Medicaid eligible (%)	0.7	0.8	0.7	0.8	
Aid and attendance receipt (%)	13.7	14.5	19.7	14.3	****
Traumatic brain injury (%)	1.5	1.6	2.5	1.6	
Dementia (%)	26.3	26.7	26.6	26.6	
Spinal cord injury (%)	2.4	3.2	6.9	3.0	****
Mean age (years) (SD)	76(12)	77(12)	74(14)	77(12)	****
Mean Elixhauser Comorbidity Index score (SD)	4.5 (3.0)	4.2 (3.0)	4.3(3.1)	4.3 (3.0)	****
Mean Nosos risk score (SD)	3.3(3.1)	3.0(3.1)	3.8(4.1)	3.2 (3.2)	****
Mean CAN score (SD)	0.12(0.15)	0.12(0.15)	0.11 (0.15)	0.12(0.15)	**
Mortality in 12-month follow-up period (%)	22.2	21.7	22.6	21.9	

** *p* < 0:05

*** *p* < 0:01

**** *p* < 0:001

**EXHIBIT 4:** Relationships among receipt of Veteran-Directed Care (VDC) and changes in hospital use and costs over time **SOURCE** Authors’ analysis of aggregated encounter and all-cause hospitalization cost data for 2015–18 from the Veterans Health Administration (VHA). **NOTES** Costs include only those attributed to an acute hospitalization in the VHA. We regressed outcomes on the indicators for receipt of VDC in the post period, indicators for receipt of other purchased care services in the post period in comparison sites without an active VDC program in fiscal year 2017, and month. In matched models, we used coarsened exact matching to create groups of recipients and nonrecipients of VDC with similar sociodemographic and clinical characteristics. Full details of the models are in the online appendix (see note 26 in text). CI is confidence interval.

	Hospital admissions		Hospitalization costs ($)	
Model	Odds ratio	95% Cl	Average incremental effect	95% Cl
**ALL-CAUSE HOSPITALIZATIONS**				
Unmatched fixed effects	0.83	(0.68, 1.02)*	−358	(−687, −29)**
Matched fixed effects	0.86	(0.75, 0.99)**	−274	(−468, −79)***
Unmatched population-averaged	0.92	(0.80, 1.06)	−149	(−452, 153)
Matched population-averaged	0.90	(0.81, 1.01)*	−163	(−325, −2)**
**AMBULATORY CARE-SENSITIVE HOSPITALIZATIONS**				
Unmatched fixed effects	1.09	(0.71, 1.39)	—^a^	—^a^
Matched fixed effects	1.15	(0.83, 1.58)	—^a^	—^a^
Unmatched population-averaged	1.03	(0.76, 1.40)	—^a^	—^a^
Matched population-averaged	1.05	(0.83, 1.33)	—^a^	—^a^

a Sparse data on ambulatory care–sensitive hospital admission costs precluded multivariable analyses.

* *p* < 0:1

** *p* < 0:05

*** *p* < 0:01
