# Supplementary material for: Users Of Veteran-Directed Care And Other Purchased Care Have Similar Hospital Use And Costs Over Time
Source: Health Aff (Millwood). Author manuscript; Available in PMC 2019 Oct 8. (PMC6781229; doi:10.1377/hlthaff.2019.00020)
Supplement: 1 [file NIHMS1040254-supplement-1.pdf]

Yuan Y, Thomas KS, Frakt AB, Pizer SD, Garrido MM. Users of veteran-directed care and other purchased care have similar hospital use and costs over time. Health Aff (Millwood). 2019;38(6).

Appendix

Active and Comparison Sites

We identified 21 sites which had an active VDC program. To be considered active, a site had to have at least 5 patients per month receiving VDC (fee purpose of visit code = 27) for at least 7 months in FY17. Comparison sites were 17 sites which had indicated interest in implementing VDC but had not yet started by FY17. Indication of interest was determined through plans to be a part of the original randomized evaluation or through personal communication with the VA Office of Geriatrics and Extended Care. A list of the 21 active sites and 17 comparison sites are shown in Exhibit A1. The other 102 VAMCs were excluded because they did not meet either of these criteria. Of these, 44 had a VDC program with at least one Veteran referred for services (https://acl.gov/programs/veteran-directed-home-and-community-based-services/veteran-directed-home-community-based) but either had low enrollment or did not distinguish between VDC and other purchased care recipients when recording service use.

Exhibit A1. Active and Comparison VHA Sites

Active VDC Program in FY17	Sta3n
**NO**	442, 508, 517, 521, 540, 581, 583, 589, 596, 603, 613, 623, 635, 644, 654, 678, 693
**YES**	405, 436, 438, 501, 516, 544, 546, 554, 556, 573, 585, 608, 648, 657, 664, 667, 671, 673, 674, 689, 692

SOURCE Authors' analysis of patient encounter data at VHA.

Inclusion/Exclusion Criteria

Veterans entered the VDC group if they were at a site with an active VDC program and had their first VDC visit recorded in FY17, regardless of other GEC service use (n = 971). Of these, we excluded 6 Veterans who received VDC before the study period in a different site (n=965). All Veterans in our VDC group had at least one inpatient or outpatient visit to the VHA in the 365 days before the index date.

We created two comparison groups. The first includes Veterans who received H/HHA, home respite, and/or contract adult day healthcare, but not VDC, at any of the sites with an active VDC program in FY17 (n = 21,313 [comparison group at active sites]). The second includes Veterans who received a purchased care service, but not VDC, at any of the 17 sites in our sample without an active VDC program (n = 15,386 [comparison group at inactive but comparable sites]). We excluded Veterans without any inpatient or outpatient visits to the VHA in the 365 days before the index date (n= 4), Veterans who received GEC services in multiple sites during the study period (n = 145), and Veterans with a death date recorded prior to the index date (n = 26). Our final sample included 37,407 Veterans: 965 VDC enrollees, 21,117 comparison individuals in active sites, and 15,325 comparison individuals in non-active sites. We were interested in hospitalizations and costs in the 12 months before and after the index date. Of our sample, 8,208 (21.9%) died before the end of the study period. As a result, our dataset included 848,516 observations (person-months).

For both intervention patients and for control patients selected from sites with active VDC programs, there is a possibility that VDC was received but coded as H/HHA prior to FY17. The stop code for VDC was not introduced

until FY17, and implementation of the code varies by site. This potential measurement error may bias our results towards the null.

Full Definitions of Variables

Cost

We calculated hospitalization costs per month for each patient. VHA data include costs on total hospitalization and are calculated upon hospital discharge. For a Veteran with a hospitalization that spanned more than one month during the study period, we divided the total hospitalization costs by length of stay, and multiplied the average daily cost by number of days in the month the Veteran was hospitalized.

Covariates

We identified a series of sociodemographic and clinical characteristic covariates to compare and match the VDC and comparison groups. The full definitions of the covariates are listed in Exhibit A2.

Exhibit A2. Full Definitions of Covariates

Variable	Definition
**Aid and Attendance**	Flag indicating increased pension from the VHA for Veterans with limitations in activities of daily living, who are bed ridden, have severely limited visual acuity, or are in a nursing home due to “mental or physical incapacity” at enrollment
**Age**	Age at service initiation date
**Male**	Gender (male; female)
**Race**	White vs non-White
**Care Assessment Need (CAN) Score (1-year mortality)**	A predictive analytic tool that estimates the relative probability of death within one year from the calculation date. CAN scores reflect percentile of risk of mortality. We used the most recent CAN score recorded prior to the index date.
**Elixhauser Comorbidity Score**	The number of Elixhauser comorbidities calculated with ICD-10 codes from any outpatient visit or inpatient stay at VHA during the 12-month baseline period
**Enrollment Priority (Group 1)**	Flag indicating Veterans with VHA-rated service-connected disabilities 50% or more disabling, or Veterans determined by VHA to be unemployable due to service-connected conditions
**Medicaid Eligibility**	Whether the patient was eligible for Medicaid. We used the most recent information available, up to the index date.
**NOSOS Risk Score**	Indicator of chronic disease burden. A risk adjustment score built on the V21 scores by adding pharmacy records as well as VA-specific items such as VA priority status and VA-computed costs
**Spinal Cord Injury (SCI)**	A flag of any paraplegia-nontraumatic, paraplegia-traumatic, quadriplegia-nontraumatic, or quadriplegia-traumatic spinal cord injury at VHA enrollment
**Traumatic Brain Injury (TBI)**	An indicator of any diagnosis of TBI during the 12-month baseline period (ICD-10 code: F07.81, S01.9X, S02.0X, S02.1X, S02.8X, S02.9X, S06.0X – S06.9X, S07.1X, S07.8X, S07.9X, S09.8X, S09.9X)
**Potential Dementia Diagnosis**	An indicator of any diagnosis of dementia during the 12-month baseline period (ICD-10 code: G30XX, G309, G3101, G3109, G311, G94, G3183, F0390, F0150, F0151, F1027, G312, F04, F0280, F0281)
**Urban/Rural Location**	Whether the patient had an urban or non-urban location

**Service Duration**	The count of months during which a patient has at least one VDC or other purchased care service each month
**Mortality**	Percentage of patients who died during the 12 months following the index date in VDC or comparison groups

SOURCE: VHA Corporate Data Warehouse (CDW) Factbooks and authors' analysis of patient data in VHA.

Differences in characteristics of enrollees in VDC and comparison groups

In Exhibit A3, we present differences in characteristics of enrollees in VDC and the comparison groups.

Exhibit A3. Characteristics of enrollees in VDC and comparison groups

Variable	Comparison group at inactive sites (N = 15,325)	Comparison group at active sites (N = 21,117)	VDC (N = 965)	Total (N = 37,407)	Significance of Comparison Test
	% or Mean (SD)	% or Mean (SD)	% or Mean (SD)	% or Mean (SD)	
**Gender: Male**	94.3	94.3	94.6	94.3	
**Race: White**	76.5	74.5	72.1	75.2	****
**Urban Location**	60.6	65.8	58.7	63.5	***
**Enrollment Priority**	15.0	14.4	19.7	14.8	****
**Medicaid Eligible**	0.7	0.8	0.7	0.8	
**Aid and Attendance**	13.7	14.5	19.7	14.3	****
**TBI**	1.5	1.6	2.5	1.6	**
**Dementia**	26.3	26.7	26.6	26.6	
**SCI**	2.4	3.2	6.9	3.0	****
**Age**	76 (12)	77 (12)	74 (14)	77 (12)	****
**Elixhauser Comorbidity Score**	4.5 (3.0)	4.2 (3.0)	4.3 (3.1)	4.3 (3.0)	****
**NOSOS Risk Score**	3.3 (3.1)	3.0 (3.1)	3.8 (4.1)	3.2 (3.2)	****
**CAN Score**	0.12 (0.15)	0.12 (0.15)	0.11 (0.15)	0.12 (0.15)	**
**Mortality in the 12 months follow-up period**	22.2	21.7	22.6	21.9	
**Any acute hospitalization in 12th month before index date**	3.7	3.3	5.0	3.5	***
**Any acute hospitalization in 6th month before index date**	4.7	4.1	5.7	4.4	***
**Any acute hospitalization in 6th month after index date**	5.0	4.6	5.0	4.8	
**Any acute hospitalization in 12th month after index date**	5.0	4.2	4.7	4.5	***

**Cost in 12th month before index date ($)**	749	752	1,624	773	****
**Cost in 6th month before index date ($)**	974	1,054	2,131	1,049	****
**Cost in 6th month after index date ($)**	1,049	1,170	1,569	1,131	*
**Cost in 12th month after index date ($)**	1,027	1,007	1,331	1,023	
	**%**	**%**	**%**	**%**	
**Any ACS acute hospitalization in 12th month before index date**	0.8	0.6	0.8	0.7	
**Any ACS acute hospitalization in 6th month before index date**	1.0	0.8	0.8	0.9	
**Any ACS acute hospitalization in 6th month after index date**	0.8	0.8	0.7	0.8	
**Any ACS acute hospitalization in 12th month after index date**	0.9	0.6	0.5	0.7	***

Source/Notes: SOURCE Authors' analysis of aggregated patient data in Veterans Health Administration. NOTE Number of missing observations for each covariate: Urban location (12), Enrollment priority (3), Medicaid eligible (6,698), Aid and attendance flag (12), Age (1), NOSOS risk score (835), Care Assessment Need (CAN) score (373). Veteran-Directed Care (VDC): Veterans who were at a site with an active VDC program and received their first VDC visit in Fiscal Year (FY)17. Comparison group at active sites: Veterans who received homemaker/home health aide (H/HHA), home respite, and/or contract adult day healthcare, but not VDC, at any of the sites with an active VDC program in FY17. Comparison group at inactive sites: Veterans who received a purchased care service, but not VDC, at any of the 17 sites in our sample without an active VDC program. ACS: ambulatory care sensitive hospital use. Chi-squared test was used to compare the difference of gender, urban location, enrollment priority, Medicaid eligible, aid and attendance, traumatic brain injury (TBI), dementia, spinal cord injury (SCI), and percent of acute hospital admissions across the three groups. Analysis of variance (ANOVA) was run to compare age, Elixhauser comorbidity, NOSOS risk score, CAN score and monthly costs across the three groups
*p < 0.1; **p < 0.05; ***p < 0.01; ****p < 0.001

Building the Difference in difference-in-differences (DDD) Model

Before building the DDD model, we compared the trends of hospital admissions and costs among VDC group, control group in sites with active VDC program and control group in sites without active VDC program (Exhibits 2 & 3 in the main text). The parallel trends assumption was not rejected.

The DDD model explicitly estimated differences among the two control groups receiving other purchased care according to whether VDC was available. That is, we estimate (VD_{post} – VD_{pre}) – ($otherGEC_{post,tmtsites}$ – $otherGEC_{pre,tmtsites}$) - ($otherGEC_{post,controlsites}$ – $otherGEC_{pre,controlsites}$), where tmtsites refers to facilities where VDC was available in FY17 and control sites refers to facilities without operational VDC programs. This is similar to a difference in difference-in-differences approach (DDD), except for the fact that ($VD_{post,tmtsites}$ – $VD_{pre,tmtsites}$) – ($VD_{post,controlsites}$ – $VD_{pre,controlsites}$) simplifies to ($VD_{post,tmtsites}$ – $VD_{pre,tmtsites}$).

We can write this model as:

$$Y = \beta_0 + \beta_1*TMT + \beta_2*TIME_t + \beta_3*OFFER + \beta_4*OFFER*TIME_t + \beta_5*TMT*TIME_t + \beta_6*OFFER*TMT + \delta*VD + u,$$

where TMT is an indicator for whether a patient belonged to VDC treatment group, OFFER is an indicator that a patient received care (either VDC or other purchase care services) at a site that offered VDC in FY17, TIME represents a set of indicators for month t, and VD is an indicator that the patient received VDC during the "post" period at a site that offered VDC.

We are interested in estimating δ. After accounting for collinearity with the treatment variable and removing terms for time-invariant characteristics; this model simplifies to:

$$Y = \beta_0 + \beta_2*TIME_t + \beta_4*OFFER*TIME_t + \delta*VD + u$$

We further simplify the $OFFER*TIME_t$ term to an indicator that equals one if the individual is in a site without VDC in the post period, and zero otherwise: NOOFFER_POST. The model is simplified to:

$$Y = \beta_0 + \beta_2*TIME_t + \beta_4*NOOFFER\_POST + \delta*VD + u.$$

Matching on Time-Invariant Covariates

Because our treatment groups differed on some sociodemographic and clinical characteristics (Exhibit A4), we explored whether matching on time-invariant covariates improved precision of estimates. We considered matching on patterns of health care use before VDC, but matching on pre-treatment levels of outcomes increases risk of bias from regression to mean. Because NOSOS risk score and CAN score are used to predict costs and risks over time and are regularly re-calculated, they also were excluded from the match. VDC and comparison groups were matched on the following characteristics: Elixhauser comorbidities, age, dementia diagnosis, and presence of either SCI or TBI. Coarsened exact matching was used to create matched subsets of the data. Analyses were re-run on matched datasets.

Exhibit A4 Standardized Differences in Covariates between VD-HCBS Treatment and Comparison Groups Before Matching

Covariates	Standardized differences			
	Between VDC and comparison group at inactive sites		Between VDC and comparison group at active sites	
	Before matching (%)	After matching using entire sample (%)	Before matching (%)	After matching using sample at active sites (%)
Gender: Male	1.5	6.7	1.2	7.3
Race: White	-10.0	-6.8	-5.3	-2.7
Urban Location	-3.8	-4.2	-14.7	-15.9
Enrollment Priority (Group 1)	12.7	8.2	14.5	8.4
Medicaid Eligible	1.0	-0.2	-1.0	-1.2
Aid and Attendance	16.2	12.7	14.0	9.3
Traumatic Brain Injury	7.0	-3.9	5.9	-3.4
Dementia	0.7	0.4	-0.2	-0.0
Spinal Cord Injury	21.6	8.7	16.9	2.2
Age	-18.9	1.9	-24.8	-0.6
Elixhauser Comorbidity Score	-9.0	-5.7	3.1	1.2
NOSOS Risk Score	14.2	9.1	21.1	12.7
CAN Score	-7.0	-0.8	-4.4	1.4
Mortality in the 12 months after the index date	0.9	4.5	2.1	6.4

SOURCE Authors' analysis of patient data in VHA. NOTE Standardized differences < 10% indicate ideal balance across groups.

Service duration and mortality

We explored whether our VDC and comparison groups differed in service duration (Exhibit A5) and mortality (Exhibit A6). VDC enrollees were more likely to receive at least one service for each month of the follow-up period than Veterans in either comparison group. Mortality rates were similar across all groups.

Exhibit A5. Distribution of personal care service duration in the 12-months after the index date

Service duration	Comparison group at inactive sites (N = 15,325)	Comparison group at active sites (N = 21,117)	VDC (N = 965)
**Months**	**%**	**%**	**%**
**1**	8.55	10.01	7.36
**2**	5.87	5.72	3.42
**3**	5.00	4.82	3.11
**4**	4.95	4.50	3.52
**5**	4.15	4.08	4.25
**6**	3.93	3.81	3.63
**7**	3.95	3.92	3.42
**8**	3.93	3.53	4.25
**9**	4.28	3.82	3.11
**10**	4.61	4.68	4.25
**11**	7.19	7.01	9.22
**12**	43.58	44.10	50.47

SOURCE Authors' analysis of patient data in VHA.

**Exhibit A6. Percentage of individuals within each comparison and treatment group who died in each month after the index date**			
**Mortality**	**Comparison group at inactive sites (N = 15,325)**	**Comparison group at active sites (N = 21,117)**	**VDC (N = 965)**
**Months**	**%**	**%**	**%**
**1**	2.09	2.33	1.97
**2**	2.09	2.25	1.97
**3**	2.20	2.09	1.97
**4**	2.12	2.02	2.07
**5**	2.03	1.90	2.07
**6**	1.82	1.80	2.28
**7**	1.67	1.76	1.97
**8**	1.77	1.77	2.18
**9**	1.72	1.49	1.76
**10**	1.57	1.49	2.07
**11**	1.50	1.44	1.66
**12**	1.61	1.38	0.62

SOURCE Authors' analysis of patient data in VHA.

Full list of models and covariates

A summary of all models and results is presented in Exhibit A7 and is followed by detailed results for specific models.

To model all-cause hospital admissions, we began with a logistic fixed effects model in our unmatched sample (Exhibit A8). Because fixed effects logistic models can be biased away from the null for panel data, we re-estimated this model with population-averaged models, which are biased towards the null (Exhibit A9). We repeated these analyses in unmatched samples within active sites (Exhibits A10 and A11). We repeated these analyses in two different matched samples: VDC and both comparison groups matched (Exhibit A12 – fixed effects, Exhibit A13 – population-averaged), and VDC and comparison group at active sites matched (Exhibit A14 – fixed effects, Exhibit A15 – population-averaged).

We analyzed cost among all individuals in our sample, and excluded one person (two person-months) with unreasonably high costs (cost > $450,000). To account for time-invariant confounders, we began with a linear fixed effects model in our unmatched sample (Exhibit A16). To better approximate the distribution of cost data,

we also ran population-averaged panel-data models with gamma distribution and log link (Exhibit A17) . We repeated our fixed effects and population-averaged models using two different matched samples (VDC and both comparison groups matched [Exhibits A18, A19], and VDC and comparison group at active sites matched [Exhibits A22, A23]). We also ran an unmatched fixed effects and population-averaged models on the sample at sites with active programs (Exhibit A20, A21). To explore whether any differences across groups might be obscured by the large number of zeroes in the dataset, we also modeled costs with a denominator of those with at least one month of non-zero cost (Exhibit A40, A41). Standard errors were calculated using bootstrapping in unmatched models.

We analyzed ambulatory care sensitive hospital admissions (Exhibit A28 – A35) using the same specifications as for models of all-cause hospital admissions.

<u>Sensitivity tests:</u>

Sensitivity tests included using a different definition of post-period, different selection criteria of VDC patients, a restricted sample of individuals who stayed alive till the end of the study, and different service durations. Most results remained non-significant. Among non-decedents, VDC receipt was associated with 15%-19% lower odds of all-cause hospital admissions, but only in our matched samples (details below). Among non-decedents, VDC receipt was associated with reduced hospitalization costs in both fixed effects and population-averaged models.

Definition of post period

To allow for potential lags in data processing, we re-ran our models with an alternate definition of the post period (beginning two months after the index date).

Sample selection criteria

In our primary analyses, we allowed VDC enrollees into the sample if they did not have any past GEC purchased care services. Our strategy allows us to model the effect of VDC, regardless of whether a patient received other GEC services in the past year, but it may introduce differences among our treatment and comparison groups. We re-ran analyses among individuals who received FY16 purchased care services in all 38 sites. Among this subset, we re-ran our models to compare treatment effect estimates to main analysis.

Results among non-decedents

In our primary analyses, we included the person-months for patients who died during the study period if the observation was for a month before the patient's month of death. Outcomes for decedents were treated as missing and non-informative. We re-ran our models for hospital admissions, restricting the sample to those who remained alive till the end of the study period. Results were substantively similar.

Service duration

We repeated our analyses on subsets of patients who received at least one service per month for at least 3 or 6 months in the follow-up period.

Results of these tests for hospital admissions are in Exhibits A24 (post period), A25 (sample selection), A50–A55 (non-decedents), and A42–A43 (service duration). Results for costs are in Exhibits A27 (post period), A28 (sample selection), A48–A49 (non-decedents), and A44–A47 (service duration).

In all exhibits below, * indicates p < .1, ** indicates p < .05, *** indicates p < .01, and **** indicates p <.001.

Exhibit A7. Summary of analytic models

Exhibit	Outcome	Cohort	Matched	Model	Effect	95%CI		Significance (p < 0.05)
A8	Use	Entire sample	No	FE logit	0.83	0.68	1.02	No
A9	Use	Entire sample	No	PA logit	0.92	0.80	1.06	No
A10	Use	Active sites	No	FE logit	0.83	0.67	1.03	No
A11	Use	Active sites	No	PA logit	0.91	0.77	1.07	No
A12	Use	Entire sample	Yes	FE logit	0.86	0.75	0.99	Yes
A13	Use	Entire sample	Yes	PA logit	0.90	0.81	1.01	No
A14	Use	Active sites	Yes	FE logit	0.88	0.76	1.01	No
A15	Use	Active sites	Yes	PA logit	0.90	0.81	1.01	No
A16	Cost	Entire sample	No	FE linear	-357.99	-687.04	-28.95	Yes
A17	Cost	Entire sample	No	PA GLM with gamma family, log link	-149.36	-452.15	153.43	No
A18	Cost	Entire sample	Yes	FE linear	-273.51	-468.46	-78.56	Yes
A19	Cost	Entire sample	Yes	PA GLM with gamma family, log link	-163.17	-324.51	-1.84	Yes
A20	Cost	Active sites	No	FE linear	-358.06	-701.17	-14.94	Yes
A21	Cost	Active sites	No	PA GLM, gamma, log	-173.11	-410.15	63.92	No
A22	Cost	Active sites	Yes	FE linear	-263.52	-465.55	-61.50	Yes
A23	Cost	Active sites	Yes	PA GLM, gamma, log	-184.03	-375.15	7.10	No
A24	Use (different post period)	Entire sample	Yes	FE logit	0.96	0.83	1.12	No
A25	Use (past GEC service)	Entire sample	Yes	FE logit	0.85	0.74	1.07	No
A26	Cost (post period)	Entire sample	Yes	FE linear	-121.10	-322.10	79.91	No
A27	Cost (GEC)	Entire sample	Yes	FE linear	-145.83	-419.79	128.12	No
A28	Use (ACS)	Entire sample	No	FE logit	1.09	0.71	1.39	No
A29	Use (ACS)	Entire sample	No	PA logit	1.03	0.76	1.40	No
A30	Use (ACS)	Entire sample	Yes	FE logit	1.15	0.83	1.58	No
A31	Use (ACS)	Entire sample	Yes	PA logit	1.05	0.83	1.33	No
A32	Use (ACS)	Active sites	No	PA logit	1.04	0.79	1.36	No
A33	Use (ACS)	Active sites	Yes	FE logit	1.15	0.83	1.59	No
A34	Use (ACS)	Active sites	Yes	PA logit	1.04	0.82	1.32	No
A35	Use (ACS)	Active sites	No	FE logit	1.09	0.75	1.57	No
A36	Use (ACS; post period)	Entire sample	Yes	FE logit	1.12	0.80	1.57	No
A37	Use (ACS; GEC)	Entire sample	Yes	FE logit	1.18	0.78	1.78	No
A38	Use (ACS; 3 mos)	Entire sample	Yes	FE logit	1.17	0.83	1.64	No
A39	Use (ACS; 6 mos)	Entire sample	Yes	FE logit	1.07	0.74	1.56	No
A40	Cost (non-zero costs only)	Entire sample	No	FE linear	-1089.57	-2169.01	-10.13	Yes
A41	Cost (non-zero costs only)	Entire sample	No	PA GLM, gamma	-348.27	-858.73	162.20	No
A42	Use (3 mos service duration)	Entire sample	Yes	FE logit	0.90	0.77	1.04	No
A43	Use (6 mos service duration)	Entire sample	Yes	FE logit	0.91	0.78	1.07	No
A44	Cost (3 mos)	Entire sample	Yes	FE linear	-215.23	-411.35	-19.11	Yes

A45	Cost (3 mos)	Entire sample	Yes	PA GLM, gamma, log	-63.68	-238.33	110.97	No
A46	Cost (6 mos)	Entire sample	Yes	FE linear	-261.81	-458.85	-64.78	Yes
A47	Cost (6 mos)	Entire sample	Yes	PA GLM, gamma, log	-42.14	-220.49	136.21	No
A48	Cost (non-decedents)	Entire sample	Yes	FE linear	-300.35	-505.09	-95.61	Yes
A49	Cost (non-decedents)	Entire sample	Yes	PA GLM, gamma, log	-212.56	-372.78	-52.35	Yes
A50	Use (non-decedents)	Entire sample	No	FE logit	0.79	0.59	1.07	No
A51	Use (non-decedents)	Entire sample	Yes	FE logit	0.81	0.68	0.95	Yes
A52	Use (non-decedents)	Active sites	Yes	FE logit	0.82	0.70	0.97	Yes
A53	Use (non-decedents)	Entire sample	No	PA logit	0.87	0.71	1.07	No
A54	Use (non-decedents)	Entire sample	Yes	PA logit	0.84	0.74	0.96	Yes
A55	Use (non-decedents)	Active sites	Yes	PA logit	0.85	0.75	0.97	Yes
A56	Use (ACS; non-decedents)	Entire sample	Yes	FE logit	1.12	0.76	1.66	No

Exhibit A8: Relationships among VDC receipt and changes in hospital use over time, entire sample, unmatched (Fixed Effects Logit Model, N = 307,916 person-months)

Variables	OR	SE	Z	95% CI	
VD	0.83	0.09	-1.78*	0.68	1.02
NO_OFFER_POST	1.12	0.04	3.10***	1.04	1.20
**month**					
**2**	1.05	0.03	1.54	0.99	1.11
**3**	1.08	0.04	2.04**	1.00	1.16
**4**	1.16	0.04	4.41****	1.09	1.24
**5**	1.19	0.04	4.93****	1.11	1.28
**6**	1.22	0.05	5.00****	1.13	1.32
**7**	1.31	0.04	7.97****	1.22	1.39
**8**	1.38	0.05	8.72****	1.28	1.48
**9**	1.56	0.06	11.03****	1.44	1.68
**10**	1.82	0.07	14.52****	1.68	1.97
**11**	2.17	0.07	23.14****	2.03	2.32
**12**	2.22	0.07	23.92****	2.08	2.37
**13**	1.17	0.05	3.95****	1.08	1.27
**14**	1.36	0.06	7.52****	1.26	1.48
**15**	1.47	0.05	10.66****	1.37	1.58
**16**	1.48	0.06	10.05****	1.37	1.60
**17**	1.48	0.06	9.12****	1.36	1.61
**18**	1.50	0.07	8.88****	1.37	1.64
**19**	1.50	0.06	9.57****	1.38	1.63
**20**	1.49	0.06	9.91****	1.38	1.62
**21**	1.48	0.06	9.51****	1.36	1.60
**22**	1.49	0.07	8.97****	1.37	1.63
**23**	1.42	0.06	7.74****	1.30	1.55
**24**	1.50	0.07	9.10****	1.37	1.63
Chi-square = 1574.76****					

Exhibit A9: Relationships among VDC receipt and changes in hospital use over time, entire sample, unmatched (Population-Averaged Logit Model, N = 848,516 person-months)

**Variable**	**OR**	**SE**	**Z**	**95% CI**	
VD	0.92	0.07	-1.21	0.80	1.06
NO_OFFER_POST	1.13	0.02	5.77****	1.08	1.18
**month**					
**2**	1.04	0.03	1.44	0.99	1.10
**3**	1.07	0.04	1.74	0.99	1.15
**4**	1.14	0.04	3.76****	1.06	1.22
**5**	1.17	0.04	4.59****	1.09	1.24
**6**	1.19	0.04	4.73****	1.11	1.28
**7**	1.26	0.04	7.17****	1.18	1.34
**8**	1.32	0.05	7.79****	1.23	1.41
**9**	1.46	0.05	11.32****	1.37	1.56
**10**	1.66	0.06	14.51****	1.55	1.78
**11**	1.92	0.07	19.29****	1.80	2.06
**12**	1.96	0.06	21.06****	1.84	2.09
**13**	1.13	0.04	3.85****	1.06	1.21
**14**	1.29	0.05	6.59****	1.19	1.38
**15**	1.37	0.05	9.04****	1.28	1.46
**16**	1.36	0.06	7.69****	1.26	1.48
**17**	1.36	0.05	8.52****	1.27	1.46
**18**	1.37	0.05	8.58****	1.27	1.47
**19**	1.36	0.06	7.29****	1.25	1.48
**20**	1.35	0.05	8.05****	1.26	1.46
**21**	1.34	0.05	7.89****	1.25	1.44
**22**	1.35	0.05	8.84****	1.26	1.44
**23**	1.29	0.05	6.86****	1.20	1.38
**24**	1.34	0.05	7.41****	1.24	1.45
**cons**	0.04	0.00	-124.18****	0.03	0.04
Chi-square = 1844****					

Exhibit A10: Relationships among VDC receipt and changes in hospital use over time (Fixed effects Logit Model; unmatched; VDC and comparison groups at active sites, N = 167,404 person-months)					
**Variable**	**OR**	**SE**	**Z**	**95% CI**	
VD	0.83	0.09	-1.66*	0.67	1.03
**month**					
**2**	1.05	0.06	0.89	0.94	1.17
**3**	1.09	0.06	1.77*	0.99	1.21
**4**	1.23	0.06	4.33****	1.12	1.36
**5**	1.21	0.06	3.87****	1.10	1.33
**6**	1.23	0.06	4.01****	1.11	1.37
**7**	1.30	0.07	4.9****	1.17	1.44
**8**	1.41	0.07	7.29****	1.29	1.55
**9**	1.63	0.07	10.92****	1.49	1.78
**10**	1.86	0.09	12.5****	1.68	2.05
**11**	2.10	0.10	15.76****	1.91	2.30
**12**	2.31	0.11	17.07****	2.10	2.55
**13**	1.22	0.07	3.32***	1.08	1.37
**14**	1.36	0.08	5.43****	1.22	1.52
**15**	1.50	0.09	6.8****	1.34	1.69
**16**	1.49	0.08	7.26****	1.34	1.65
**17**	1.47	0.07	7.54****	1.33	1.62
**18**	1.59	0.09	8.53****	1.43	1.76
**19**	1.50	0.08	7.52****	1.35	1.67
**20**	1.51	0.08	7.43****	1.35	1.68
**21**	1.52	0.09	7.36****	1.36	1.70
**22**	1.52	0.08	7.83****	1.37	1.69
**23**	1.41	0.07	6.57****	1.28	1.57
**24**	1.51	0.08	7.36****	1.35	1.68
Chi-square = 882.36****					

Exhibit A11: Relationships among VDC receipt and changes in hospital use over time (Population-Averaged Logit Model; unmatched; VDC and comparison groups at active sites, N = 500,788 person-months)

Variable	OR	SE	Z	95% CI	
VD	0.91	0.08	-1.11	0.77	1.07
**month**					
**2**	1.04	0.04	1.03	0.96	1.13
**3**	1.08	0.04	2.09**	1.00	1.16
**4**	1.20	0.05	4.4****	1.11	1.30
**5**	1.18	0.05	3.5****	1.07	1.29
**6**	1.20	0.05	4.67****	1.11	1.29
**7**	1.25	0.06	5.02****	1.15	1.36
**8**	1.34	0.06	6.21****	1.22	1.47
**9**	1.51	0.07	9.05****	1.38	1.65
**10**	1.68	0.07	12.02****	1.54	1.83
**11**	1.85	0.08	15.14****	1.71	2.01
**12**	2.00	0.10	14.2****	1.82	2.20
**13**	1.18	0.06	3.25***	1.07	1.30
**14**	1.29	0.07	4.89****	1.17	1.43
**15**	1.40	0.06	7.35****	1.28	1.53
**16**	1.38	0.07	6.49****	1.25	1.52
**17**	1.36	0.07	6.27****	1.23	1.49
**18**	1.45	0.06	8.87****	1.33	1.57
**19**	1.38	0.07	6.34****	1.25	1.52
**20**	1.38	0.07	6.7****	1.26	1.52
**21**	1.38	0.08	5.99****	1.24	1.54
**22**	1.38	0.07	6.64****	1.26	1.52
**23**	1.30	0.06	5.31****	1.18	1.43
**24**	1.37	0.08	5.68****	1.23	1.52
**cons**	0.03	0.00	-94.2****	0.03	0.04

Chi-square = 985.37****

Exhibit A12: Relationships among VDC receipt and changes in hospital use over time (Fixed Effects Logit Model, matched VCD and comparison groups across all sites; N = 305,588 person-months)

Variable	OR	SE	Z	95% CI	
VD	0.86	0.06	-2.03**	0.75	0.99
NO_OFFER_POST	1.07	0.02	3.02***	1.02	1.12
**Month**					
**2**	1.04	0.04	0.98	0.96	1.12
**3**	1.05	0.04	1.32	0.97	1.14
**4**	1.15	0.04	3.52****	1.06	1.24
**5**	1.20	0.05	4.71****	1.11	1.30
**6**	1.19	0.05	4.53****	1.11	1.29
**7**	1.26	0.05	6.04****	1.17	1.36
**8**	1.37	0.05	8.19****	1.27	1.47
**9**	1.54	0.06	11.46****	1.43	1.65
**10**	1.80	0.07	15.99****	1.67	1.93
**11**	2.06	0.07	20.01****	1.92	2.21
**12**	2.07	0.07	20.04****	1.92	2.22
**13**	1.08	0.04	1.80*	0.99	1.17
**14**	1.28	0.05	6.15****	1.18	1.38
**15**	1.39	0.06	8.31****	1.29	1.50
**16**	1.45	0.06	9.42****	1.35	1.57
**17**	1.40	0.06	8.40****	1.30	1.52
**18**	1.40	0.06	8.25****	1.29	1.51
**19**	1.43	0.06	8.81****	1.32	1.55
**20**	1.41	0.06	8.39****	1.30	1.53
**21**	1.38	0.06	7.86****	1.28	1.50
**22**	1.39	0.06	7.91****	1.28	1.51
**23**	1.29	0.05	6.08****	1.19	1.40
**24**	1.40	0.06	8.05****	1.29	1.52
Chi-square = 1317.73****					

Exhibit A13: Relationships among VDC receipt and changes in hospital use over time (Population-averaged logit model, matched VDC and comparison groups across all sites; N = 843,516 person-months)

Variable	OR	SE	Z	95% CI	
VD	0.90	0.05	-1.82*	0.81	1.01
NO_OFFER_POST	1.07	0.02	4.07****	1.04	1.11
**month**					
**2**	1.03	0.03	0.99	0.97	1.10
**3**	1.05	0.04	1.33	0.98	1.12
**4**	1.12	0.04	3.55****	1.05	1.20
**5**	1.17	0.04	4.74****	1.10	1.25
**6**	1.16	0.04	4.56****	1.09	1.24
**7**	1.22	0.04	6.07****	1.14	1.30
**8**	1.30	0.04	8.22****	1.22	1.39
**9**	1.43	0.05	11.45****	1.35	1.53
**10**	1.63	0.05	15.89****	1.54	1.73
**11**	1.82	0.06	19.76****	1.72	1.93
**12**	1.82	0.06	19.80****	1.72	1.93
**13**	1.06	0.04	1.69*	0.99	1.13
**14**	1.22	0.04	6.00****	1.14	1.30
**15**	1.31	0.04	8.09****	1.23	1.39
**16**	1.35	0.04	9.09****	1.27	1.44
**17**	1.31	0.04	8.00****	1.22	1.39
**18**	1.30	0.04	7.78****	1.22	1.39
**19**	1.32	0.04	8.28****	1.24	1.41
**20**	1.30	0.04	7.83****	1.22	1.39
**21**	1.28	0.04	7.25****	1.20	1.37
**22**	1.28	0.04	7.27****	1.20	1.37
**23**	1.21	0.04	5.43****	1.13	1.29
**24**	1.29	0.04	7.35****	1.20	1.38
**cons**	0.04	0.00	-120.44****	0.04	0.04

Chi-square = 1301.89****

**Exhibit A14: Relationships among VDC receipt and changes in hospital use over time (Fixed Effects Logit Model, matched VDC and comparison group at active sites; N = 165,988 person-months)**					
**Variable**	**OR**	**SE**	**Z**	**95% CI**	
VD	0.88	0.06	-1.82*	0.76	1.01
**month**					
**2**	1.04	0.05	0.72	0.94	1.15
**3**	1.06	0.05	1.07	0.96	1.17
**4**	1.19	0.06	3.44***	1.08	1.32
**5**	1.20	0.06	3.53****	1.08	1.32
**6**	1.19	0.06	3.37***	1.07	1.31
**7**	1.23	0.06	4.15****	1.12	1.36
**8**	1.36	0.07	6.11****	1.23	1.50
**9**	1.59	0.08	9.49****	1.44	1.75
**10**	1.79	0.09	12.14****	1.63	1.97
**11**	1.92	0.09	13.61****	1.74	2.10
**12**	2.13	0.10	16.01****	1.94	2.34
**13**	1.08	0.06	1.49	0.98	1.20
**14**	1.27	0.06	4.68****	1.15	1.40
**15**	1.41	0.07	6.81****	1.28	1.56
**16**	1.43	0.07	7.06****	1.30	1.58
**17**	1.31	0.07	5.25****	1.19	1.45
**18**	1.40	0.07	6.56****	1.27	1.55
**19**	1.39	0.07	6.38****	1.26	1.54
**20**	1.43	0.07	6.81****	1.29	1.58
**21**	1.35	0.07	5.73****	1.22	1.50
**22**	1.37	0.07	5.99****	1.24	1.52
**23**	1.21	0.07	3.46***	1.09	1.34
**24**	1.35	0.07	5.51****	1.21	1.50
Chi-square = 739.21****					

Exhibit A15: Relationships among VDC receipt and changes in hospital use over time (Population-averaged logit model, matched VDC and comparison at active sites; N = 497,651 person-months)

Variable	OR	SE	Z	95% CI	
VD	0.90	0.05	-1.82*	0.81	1.01
**month**					
**2**	1.03	0.04	0.73	0.95	1.12
**3**	1.05	0.05	1.08	0.96	1.14
**4**	1.16	0.05	3.46***	1.07	1.26
**5**	1.16	0.05	3.55****	1.07	1.26
**6**	1.15	0.05	3.39***	1.06	1.25
**7**	1.19	0.05	4.17****	1.10	1.29
**8**	1.29	0.05	6.12****	1.19	1.40
**9**	1.47	0.06	9.46****	1.35	1.59
**10**	1.62	0.06	12.04****	1.49	1.75
**11**	1.70	0.07	13.46****	1.58	1.84
**12**	1.85	0.07	15.76****	1.71	2.00
**13**	1.07	0.05	1.49	0.98	1.16
**14**	1.22	0.05	4.63****	1.12	1.32
**15**	1.32	0.06	6.69****	1.22	1.44
**16**	1.33	0.06	6.87****	1.23	1.45
**17**	1.24	0.05	5.04****	1.14	1.35
**18**	1.31	0.06	6.31****	1.20	1.42
**19**	1.30	0.06	6.1****	1.19	1.41
**20**	1.32	0.06	6.5****	1.21	1.44
**21**	1.26	0.05	5.39****	1.16	1.38
**22**	1.28	0.06	5.63****	1.17	1.39
**23**	1.15	0.05	3.12***	1.05	1.25
**24**	1.25	0.06	5.13****	1.15	1.37
**cons**	0.04	0.00	-92.63****	0.04	0.05
Chi-square = 739.16****					

**Exhibit A16: Relationships among VDC receipt and changes in acute care costs over time (Fixed Effects Linear Regression Model; unmatched; denominator: entire sample; N = 848,490 person-months)**					
**Variable**	**Coef.**	**SE**	**Z**	**95% CI**	
**VD**	-357.99	167.88	-2.13**	-687.04	-28.95
**NO_OFFER_POST**	87.82	48.68	1.8*	-7.59	183.22
**month**					
**2**	33.07	33.70	0.98	-32.98	99.12
**3**	30.39	39.83	0.76	-47.67	108.45
**4**	102.93	36.42	2.83***	31.55	174.32
**5**	153.68	45.30	3.39***	64.90	242.46
**6**	184.39	43.65	4.22****	98.83	269.94
**7**	276.21	40.51	6.82****	196.82	355.60
**8**	268.98	43.31	6.21****	184.10	353.85
**9**	475.57	61.01	7.8****	356.00	595.14
**10**	689.59	49.44	13.95****	592.69	786.49
**11**	835.42	49.06	17.03****	739.27	931.57
**12**	617.65	45.57	13.55****	528.34	706.97
**13**	64.11	47.23	1.36*	-28.45	156.67
**14**	267.69	52.45	5.1****	164.89	370.48
**15**	292.21	53.66	5.45****	187.04	397.39
**16**	364.00	60.81	5.99****	244.82	483.18
**17**	404.48	58.94	6.86****	288.96	520.00
**18**	406.18	58.73	6.92****	291.07	521.28
**19**	382.67	48.35	7.91****	287.91	477.43
**20**	447.79	46.95	9.54****	355.77	539.82
**21**	396.58	53.48	7.42****	291.76	501.39
**22**	435.51	54.17	8.04****	329.33	541.69
**23**	346.27	52.12	6.64****	244.13	448.42
**24**	370.90	57.20	6.48****	258.79	483.02
**_cons**	738.86	26.40	27.99****	687.12	790.61
Chi-square = 973.02****					

**Exhibit A17: Relationships among VDC receipt and changes in acute care costs over time (Population-averaged GLM Model with gamma distribution; unmatched; denominator: entire sample; N = 848,514 person-months)**					
**Variable**	**Average Incremental Effect**	**Std. Err.**	**z**	**95% C. I.**	
**VD**	-149.36	154.49	-0.97	-452.15	153.43
**NO_OFFER_POST**	67.90	37.60	1.81*	-5.80	141.60
	**Coef.**	**SE**	**Z**	**95% CI**	
**VD**	-0.15	0.16	-0.9	-0.46	0.17
**NO_OFFER_POST**	0.06	0.03	1.84*	0.00	0.13
**month**					
**2**	0.04	0.04	1.1	-0.03	0.12
**3**	0.04	0.04	0.89*	-0.05	0.12
**4**	0.12	0.05	2.69***	0.03	0.22
**5**	0.18	0.04	4.03****	0.09	0.27
**6**	0.21	0.05	4.24****	0.11	0.31
**7**	0.31	0.04	7.31****	0.22	0.39
**8**	0.30	0.04	7.34****	0.22	0.38
**9**	0.48	0.04	11.29****	0.40	0.56
**10**	0.64	0.04	17.14****	0.56	0.71
**11**	0.73	0.04	18.84****	0.66	0.81
**12**	0.59	0.04	15.06****	0.51	0.66
**13**	0.09	0.06	1.5	-0.03	0.21
**14**	0.30	0.05	6.13****	0.20	0.39
**15**	0.32	0.05	6.08****	0.22	0.42
**16**	0.38	0.06	6.51****	0.26	0.49
**17**	0.41	0.05	8.64****	0.32	0.50
**18**	0.41	0.04	9.58****	0.32	0.49
**19**	0.38	0.05	7.73****	0.29	0.48
**20**	0.44	0.05	8.13****	0.33	0.55
**21**	0.39	0.05	7.87****	0.30	0.49
**22**	0.43	0.05	7.84****	0.32	0.53
**23**	0.34	0.05	7.14****	0.25	0.44
**24**	0.36	0.05	7.14****	0.26	0.46
**_cons**	6.65	0.04	182.22****	6.58	6.72
Chi-square = 1171.83****					

Exhibit A18: Relationships among VDC receipt and changes in acute care costs over time (Fixed Effects Linear Regression Model; matched VDC and comparison groups across all sites; denominator: entire sample; N = 843,514 person-months)

Variable	Coef.	SE	t	95% CI	
**VD**	-273.51	99.47	-2.75***	-468.46	-78.56
**NO_OFFER_POST**	95.20	32.08	2.97***	32.32	158.08
**month**					
**2**	34.41	51.96	0.66	-67.44	136.25
**3**	11.75	51.96	0.23	-90.09	113.59
**4**	86.68	51.96	1.67	-15.16	188.52
**5**	152.33	51.96	2.93***	50.49	254.17
**6**	190.65	51.96	3.67****	88.81	292.49
**7**	285.83	51.96	5.5****	183.98	387.67
**8**	325.09	51.96	6.26****	223.25	426.93
**9**	551.62	51.96	10.62****	449.78	653.46
**10**	803.45	51.96	15.46****	701.60	905.29
**11**	818.82	51.96	15.76****	716.98	920.67
**12**	594.18	51.96	11.44****	492.34	696.02
**13**	-57.26	53.73	-1.07	-162.56	48.04
**14**	165.50	54.01	3.06****	59.65	271.36
**15**	213.54	54.31	3.93****	107.10	319.98
**16**	341.11	54.59	6.25****	234.11	448.11
**17**	342.09	54.88	6.23****	234.54	449.65
**18**	348.14	55.16	6.31****	240.03	456.25
**19**	362.55	55.43	6.54****	253.91	471.18
**20**	381.32	55.69	6.85****	272.17	490.48
**21**	348.78	55.97	6.23****	239.08	458.49
**22**	345.71	56.23	6.15****	235.50	455.92
**23**	251.91	56.48	4.46****	141.21	362.61
**24**	320.33	56.74	5.65****	209.12	431.54
**_cons**	949.10	36.76	25.82****	877.05	1021.15
F = 35.05****					

Exhibit A19: Relationships among VDC receipt and changes in acute care costs over time (Population-averaged GLM Model with gamma distribution; matched; denominator: entire sample; N = 843,538 person-months)

Variable	Average Incremental Effect	Std. Err.	z	95% C. I.	
**VD**	-163.17	82.32	-1.98**	-324.51	-1.84
**NO_OFFER_POST**	55.44	31.09	1.78*	-5.50	116.37
	**Coef.**	**SE**	**Z**	**95% CI**	
**VD**	-0.14	0.07	-1.86*	-0.28	0.01
**NO_OFFER_POST**	0.04	0.02	1.81*	0.00	0.09
**month**					
**2**	0.03	0.04	0.84	-0.05	0.11
**3**	0.01	0.04	0.29	-0.07	0.09
**4**	0.08	0.04	2.07**	0.00	0.17
**5**	0.14	0.04	3.53****	0.06	0.22
**6**	0.18	0.04	4.35****	0.10	0.26
**7**	0.26	0.04	6.26****	0.18	0.34
**8**	0.29	0.04	7****	0.21	0.37
**9**	0.45	0.04	10.92****	0.37	0.53
**10**	0.60	0.04	14.64****	0.52	0.68
**11**	0.61	0.04	14.85****	0.53	0.69
**12**	0.47	0.04	11.59****	0.39	0.55
**13**	-0.04	0.04	-0.96	-0.12	0.04
**14**	0.17	0.04	3.92****	0.08	0.25
**15**	0.21	0.04	4.84****	0.12	0.29
**16**	0.31	0.04	7.17****	0.22	0.39
**17**	0.30	0.04	7.06****	0.22	0.39
**18**	0.31	0.04	7.1****	0.22	0.39
**19**	0.32	0.04	7.25****	0.23	0.40
**20**	0.33	0.04	7.57****	0.24	0.42
**21**	0.30	0.04	6.91****	0.22	0.39
**22**	0.30	0.04	6.75****	0.21	0.38
**23**	0.22	0.04	4.91****	0.13	0.30
**24**	0.28	0.04	6.19****	0.19	0.36
**_cons**	6.89	0.03	216.03****	6.82	6.95

Chi-square = 800.79****

Exhibit A20: Relationships among VDC receipt and changes in acute care costs over time (Fixed Effects Linear Regression Model; unmatched sample at active sites; N = 500,800 person-months)					
**Variable**	**Coef.**	**SE**	**Z**	**95% CI**	
**VD**	-358.06	175.06	-2.05**	-701.17	-14.94
**month**					
**2**	17.30	38.33	0.45	-57.82	92.42
**3**	24.35	40.66	0.6	-55.35	104.05
**4**	142.82	47.66	3***	49.42	236.23
**5**	176.96	50.68	3.49****	77.64	276.29
**6**	210.88	54.85	3.84****	103.36	318.39
**7**	311.14	69.48	4.48****	174.97	447.31
**8**	299.78	70.02	4.28****	162.54	437.01
**9**	525.17	74.56	7.04****	379.03	671.30
**10**	701.62	65.18	10.76****	573.86	829.37
**11**	811.64	72.08	11.26****	670.37	952.91
**12**	658.45	68.87	9.56****	523.46	793.44
**13**	62.44	65.05	0.96	-65.06	189.94
**14**	242.86	51.66	4.7****	141.61	344.11
**15**	294.68	62.18	4.74****	172.81	416.55
**16**	399.24	60.87	6.56****	279.93	518.55
**17**	448.41	69.78	6.43****	311.64	585.18
**18**	482.90	64.42	7.5****	356.64	609.16
**19**	390.94	62.90	6.22****	267.66	514.22
**20**	474.88	71.62	6.63****	334.51	615.26
**21**	418.05	62.80	6.66****	294.96	541.14
**22**	417.90	62.34	6.7****	295.71	540.09
**23**	376.25	64.36	5.85****	250.12	502.38
**24**	390.14	67.71	5.76****	257.42	522.86
**_cons**	756.76	43.85	17.26****	670.81	842.72
Chi-square = 663.75****					

**Exhibit A21: Relationships among VDC receipt and changes in acute care costs over time (Population-averaged GLM Model with gamma distribution; unmatched; denominator: sample at active sites; N = 500,800 person-months)**					
**Variable**	**Average Incremental Effect**	**Std. Err.**	**z**	**95% C. I.**	
**VD**	-173.11	120.94	-1.43	-410.15	63.92
	**Coef.**	**SE**	**Z**	**95% CI**	
**VD**	-0.15	0.11	-1.43	-0.37	0.06
**month**					
**2**	0.02	0.05	0.45	-0.07	0.12
**3**	0.03	0.05	0.59	-0.07	0.13
**4**	0.17	0.06	2.91***	0.05	0.28
**5**	0.20	0.06	3.44***	0.09	0.32
**6**	0.24	0.06	3.81****	0.11	0.36
**7**	0.33	0.07	4.56****	0.19	0.47
**8**	0.32	0.08	4.27****	0.17	0.47
**9**	0.51	0.07	6.83****	0.36	0.66
**10**	0.64	0.06	10.16****	0.51	0.76
**11**	0.71	0.06	10.96****	0.58	0.83
**12**	0.61	0.07	8.94****	0.47	0.74
**13**	0.06	0.08	0.8	-0.09	0.22
**14**	0.26	0.06	4.18****	0.14	0.38
**15**	0.30	0.07	4.34****	0.17	0.44
**16**	0.40	0.06	6.36****	0.28	0.52
**17**	0.44	0.07	5.99****	0.29	0.58
**18**	0.47	0.07	6.86****	0.33	0.60
**19**	0.38	0.07	5.57****	0.25	0.51
**20**	0.46	0.08	6****	0.31	0.61
**21**	0.40	0.07	5.81****	0.27	0.54
**22**	0.40	0.06	6.25****	0.27	0.52
**23**	0.35	0.07	4.81****	0.21	0.50
**24**	0.37	0.07	5.03****	0.23	0.51
**_cons**	6.67	0.06	116.78****	6.56	6.78
Chi-square = 669.55****					

Exhibit A22: Relationships among VDC receipt and changes in acute care costs over time (Fixed Effects Linear Regression Model; matched VDC and comparison groups; denominator: sample at active sites N = 497,649 person-months)

Variable	Coef.	SE	t	95% CI	
**VD**	-263.52	103.08	-2.56**	-465.55	-61.50
**month**					
**2**	-12.54	70.09	-0.18	-149.92	124.83
**3**	-18.94	70.09	-0.27	-156.31	118.43
**4**	104.25	70.09	1.49	-33.12	241.62
**5**	181.86	70.09	2.59***	44.49	319.23
**6**	205.63	70.09	2.93***	68.26	343.00
**7**	331.75	70.09	4.73****	194.38	469.13
**8**	329.43	70.09	4.7****	192.06	466.80
**9**	603.80	70.09	8.61****	466.43	741.17
**10**	817.21	70.09	11.66****	679.84	954.58
**11**	760.20	70.09	10.85****	622.82	897.57
**12**	663.14	70.09	9.46****	525.77	800.51
**13**	-86.91	70.24	-1.24	-224.57	50.75
**14**	114.15	70.64	1.62	-24.32	252.61
**15**	195.48	71.07	2.75***	56.19	334.77
**16**	376.00	71.46	5.26****	235.95	516.05
**17**	370.42	71.84	5.16****	229.62	511.23
**18**	378.94	72.23	5.25****	237.37	520.50
**19**	361.53	72.60	4.98****	219.24	503.81
**20**	392.71	72.97	5.38****	249.70	535.72
**21**	363.05	73.36	4.95****	219.27	506.83
**22**	319.94	73.70	4.34****	175.49	464.40
**23**	260.59	74.05	3.52****	115.46	405.73
**24**	320.05	74.40	4.3****	174.23	465.86
**_cons**	1029.48	49.59	20.76****	932.29	1126.67

F = 22.14****

Exhibit A23: Relationships among VDC receipt and changes in acute care costs over time (Population-averaged GLM Model with gamma distribution; matched; denominator: sample at active sites; N = 497,673 person-months)

Variable	Average Incremental Effect	Std. Err.	z	95% C. I.	
VD	-184.03	97.51	-1.89*	-375.15	7.10
	Coef.	SE	Z	95% CI	
VD	-0.14	0.07	-1.89*	-0.28	0.01
month					
2	-0.01	0.05	-0.23	-0.11	0.09
3	-0.02	0.05	-0.34	-0.12	0.08
4	0.09	0.05	1.79*	-0.01	0.20
5	0.16	0.05	3.02***	0.06	0.26
6	0.18	0.05	3.39***	0.07	0.28
7	0.27	0.05	5.2****	0.17	0.38
8	0.27	0.05	5.17****	0.17	0.37
9	0.45	0.05	8.61****	0.35	0.55
10	0.57	0.05	10.91****	0.47	0.67
11	0.54	0.05	10.33****	0.44	0.64
12	0.49	0.05	9.28****	0.38	0.59
13	-0.09	0.05	-1.76*	-0.20	0.01
14	0.09	0.05	1.78*	-0.01	0.20
15	0.16	0.05	3.04***	0.06	0.27
16	0.30	0.05	5.61****	0.20	0.40
17	0.29	0.05	5.46****	0.19	0.40
18	0.30	0.05	5.55****	0.19	0.41
19	0.28	0.05	5.23****	0.18	0.39
20	0.31	0.05	5.66****	0.20	0.42
21	0.28	0.05	5.17****	0.18	0.39
22	0.25	0.06	4.5****	0.14	0.36
23	0.20	0.06	3.55****	0.09	0.30
24	0.25	0.06	4.44****	0.14	0.36
_cons	6.97	0.04	169.44****	6.88	7.05

Chi-square = 501.92****

Exhibit A24: Relationships among VDC receipt and changes in hospital use over time, with post period beginning 2 months after the index date (Fixed Effects Model, matched VCD and comparison groups across all sites; N = 305,588 person-months)

Variables	OR	SE	Z	95% CI	
VD	0.96	0.07	-0.49	0.83	1.12
NO_OFFER_POST	1.07	0.02	3.08***	1.03	1.12
**month**					
**2**	1.04	0.04	0.98	0.96	1.12
**3**	1.05	0.04	1.32	0.97	1.14
**4**	1.15	0.04	3.52****	1.06	1.24
**5**	1.20	0.05	4.71****	1.11	1.30
**6**	1.19	0.05	4.53****	1.11	1.29
**7**	1.26	0.05	6.04****	1.17	1.36
**8**	1.37	0.05	8.19****	1.27	1.47
**9**	1.54	0.06	11.46****	1.43	1.65
**10**	1.80	0.07	15.99****	1.67	1.93
**11**	2.06	0.07	20.01****	1.92	2.21
**12**	2.06	0.07	20.04****	1.92	2.22
**13**	1.10	0.04	2.51**	1.02	1.19
**14**	1.31	0.05	7.02****	1.22	1.41
**15**	1.38	0.06	8.19****	1.28	1.50
**16**	1.45	0.06	9.29****	1.34	1.57
**17**	1.40	0.06	8.27****	1.29	1.51
**18**	1.39	0.06	8.13****	1.28	1.51
**19**	1.42	0.06	8.69****	1.32	1.54
**20**	1.40	0.06	8.27****	1.30	1.52
**21**	1.38	0.06	7.74****	1.27	1.49
**22**	1.38	0.06	7.8****	1.27	1.50
**23**	1.29	0.05	5.97****	1.18	1.40
**24**	1.40	0.06	7.94****	1.29	1.52

Chi-square = 1312.85****

**Exhibit A25: Relationships among VDC receipt and changes in hospital use over time, with VDC group restricted to those who received past GEC purchased care services (Fixed Effects Model, matched VCD and comparison groups across all sites; N = 298,486 person-months)**					
**Variables**	**OR**	**SE**	**Z**	**95% CI**	
VD	0.85	0.11	-1.26	0.74	1.07
NO_OFFER_POST	1.12	0.04	2.02**	1.00	1.09
**month**					
**2**	1.04	0.04	1.11	0.97	1.13
**3**	1.07	0.04	1.71*	0.99	1.15
**4**	1.13	0.04	3.3***	1.05	1.22
**5**	1.20	0.05	4.89****	1.12	1.30
**6**	1.19	0.04	4.48****	1.10	1.28
**7**	1.23	0.05	5.44****	1.14	1.32
**8**	1.29	0.05	6.89****	1.20	1.39
**9**	1.48	0.05	10.66****	1.38	1.59
**10**	1.77	0.06	15.88****	1.65	1.90
**11**	2.01	0.07	19.79****	1.88	2.16
**12**	2.04	0.07	20.11****	1.90	2.18
**13**	1.05	0.04	1.23	0.97	1.14
**14**	1.25	0.05	5.8****	1.16	1.35
**15**	1.34	0.05	7.51****	1.24	1.45
**16**	1.42	0.06	9.05****	1.32	1.53
**17**	1.36	0.05	7.72****	1.25	1.46
**18**	1.34	0.05	7.42****	1.24	1.45
**19**	1.40	0.06	8.53****	1.30	1.52
**20**	1.37	0.05	7.8****	1.26	1.48
**21**	1.35	0.05	7.38****	1.25	1.46
**22**	1.36	0.06	7.63****	1.26	1.48
**23**	1.29	0.05	6.15***	1.19	1.40
**24**	1.40	0.06	8.25****	1.29	1.52
Chi-square = 1284.36****					

Exhibit A26: Relationships among VDC receipt and changes in acute care costs over time, with post period beginning 2 months after the index date (Fixed Effects Linear Regression Model; matched VDC and comparison groups across all sites; N = 843,538 person-months)

Variable	Coef.	SE	t	95% CI	
**VD**	-121.10	102.55	-1.18	-322.10	79.91
**NO_OFFER_POST**	47.21	33.03	1.43	-17.52	111.95
**month**					
**2**	34.41	51.96	0.66	-67.43	136.25
**3**	11.76	51.96	0.23	-90.08	113.60
**4**	86.68	51.96	1.67*	-15.16	188.52
**5**	152.33	51.96	2.93***	50.49	254.17
**6**	190.65	51.96	3.67****	88.81	292.49
**7**	285.83	51.96	5.5****	183.99	387.67
**8**	325.09	51.96	6.26****	223.25	426.93
**9**	551.62	51.96	10.62****	449.78	653.46
**10**	803.44	51.96	15.46****	701.60	905.28
**11**	818.82	51.96	15.76****	716.98	920.66
**12**	594.18	51.96	11.44****	492.34	696.02
**13**	-25.58	51.96	-0.49	-127.42	76.26
**14**	197.22	52.25	3.77****	94.81	299.63
**15**	229.16	54.41	4.21****	122.51	335.80
**16**	356.71	54.69	6.52****	249.51	463.91
**17**	357.69	54.98	6.51****	249.94	465.44
**18**	363.73	55.26	6.58****	255.43	472.04
**19**	378.15	55.53	6.81****	269.32	486.98
**20**	396.94	55.79	7.11****	287.60	506.29
**21**	364.42	56.07	6.5****	254.53	474.32
**22**	361.34	56.33	6.42****	250.94	471.74
**23**	267.56	56.58	4.73****	156.66	378.45
**24**	335.98	56.84	5.91****	224.58	447.37
**_cons**	949.10	36.76	25.82****	877.05	1021.15

F = 34.45****

Exhibit A27: Relationships among VDC receipt and changes in acute care costs over time, with VDC group restricted to those who received past GEC purchased care services (Fixed Effects Linear Regression Model; matched VDC and comparison groups across all sites; N = 825,621 person-months)

Variable	Coef.	SE	t	95% CI	
**VD**	-145.83	139.78	-1.04	-419.79	128.12
**NO_OFFER_POST**	60.00	33.43	1.79*	-5.51	125.51
**month**					
**2**	60.39	54.40	1.11	-46.23	167.00
**3**	2.54	54.40	0.05	-104.08	109.16
**4**	68.42	54.40	1.26	-38.19	175.04
**5**	141.85	54.40	2.61***	35.23	248.46
**6**	173.15	54.40	3.18***	66.53	279.76
**7**	239.31	54.40	4.4****	132.70	345.93
**8**	256.50	54.40	4.72****	149.88	363.12
**9**	493.28	54.40	9.07****	386.66	599.90
**10**	793.72	54.40	14.59****	687.10	900.34
**11**	830.36	54.40	15.26****	723.74	936.97
**12**	611.20	54.40	11.24****	504.58	717.82
**13**	-93.46	56.20	-1.66*	-203.61	16.69
**14**	155.31	56.50	2.75***	44.56	266.05
**15**	184.90	56.82	3.25****	73.54	296.26
**16**	332.09	57.12	5.81****	220.13	444.05
**17**	296.65	57.44	5.16****	184.08	409.23
**18**	324.05	57.75	5.61****	210.87	437.23
**19**	336.16	58.03	5.79****	222.41	449.90
**20**	343.28	58.32	5.89****	228.98	457.57
**21**	359.24	58.62	6.13****	244.34	474.14
**22**	330.60	58.90	5.61****	215.14	446.05
**23**	255.32	59.18	4.31****	139.32	371.31
**24**	330.52	59.46	5.56****	213.97	447.07
**_cons**	1060.83	38.49	27.56****	985.39	1136.26

F = 31.99****

Exhibit A28: Relationships among VDC receipt and changes in ambulatory care sensitive - hospital use over time, entire sample, unmatched (Fixed Effects Logit Model, N = 97,732 person-months)

Variables	OR	SE	Z	95% CI	
VD	1.09	0.20	0.45	0.71	1.39
NO_OFFER_POST	1.13	0.07	2.1**	1.05	1.31
**month**					
**2**	1.11	0.09	1.18	0.94	1.30
**3**	1.18	0.09	2.05**	1.01	1.37
**4**	1.35	0.12	3.32***	1.13	1.62
**5**	1.39	0.12	3.7****	1.17	1.66
**6**	1.37	0.11	3.78****	1.16	1.61
**7**	1.37	0.11	3.99****	1.17	1.59
**8**	1.36	0.13	3.28***	1.13	1.63
**9**	1.49	0.13	4.6****	1.26	1.77
**10**	1.64	0.13	6.26****	1.41	1.92
**11**	2.12	0.17	9.25****	1.81	2.48
**12**	2.31	0.20	9.78****	1.95	2.73
**13**	1.36	0.13	3.34***	1.14	1.64
**14**	1.53	0.14	4.8****	1.29	1.83
**15**	1.67	0.17	5.02****	1.36	2.03
**16**	1.61	0.18	4.24****	1.29	2.01
**17**	1.53	0.16	4.19****	1.26	1.87
**18**	1.45	0.14	3.92****	1.20	1.75
**19**	1.58	0.16	4.35****	1.28	1.93
**20**	1.61	0.16	4.72****	1.32	1.95
**21**	1.56	0.16	4.32****	1.28	1.91
**22**	1.55	0.18	3.72****	1.23	1.95
**23**	1.45	0.17	3.19***	1.16	1.83
**24**	1.65	0.17	4.75****	1.34	2.03
Chi-square = 488.75****					

Exhibit A29: Relationships among VDC receipt and changes in ambulatory care sensitive - hospital use over time, entire sample, unmatched (Population-Averaged Logit Model, N = 848,516 person-months)

Variable	OR	SE	Z	95% CI	
VD	1.03	0.16	0.21	0.76	1.40
NO_OFFER_POST	1.23	0.06	4.37****	1.12	1.36
**month**					
**2**	1.10	0.09	1.18	0.94	1.28
**3**	1.16	0.08	2.18**	1.02	1.33
**4**	1.32	0.10	3.61****	1.14	1.54
**5**	1.36	0.11	3.79****	1.16	1.59
**6**	1.34	0.11	3.6****	1.14	1.57
**7**	1.33	0.10	3.95****	1.16	1.54
**8**	1.33	0.10	3.84****	1.15	1.53
**9**	1.45	0.11	4.99****	1.25	1.68
**10**	1.58	0.11	6.76****	1.38	1.81
**11**	1.98	0.15	9.31****	1.72	2.29
**12**	2.14	0.15	10.9****	1.87	2.46
**13**	1.28	0.10	3.09***	1.09	1.50
**14**	1.41	0.11	4.46****	1.21	1.65
**15**	1.51	0.12	5.45****	1.30	1.76
**16**	1.45	0.11	5.05****	1.26	1.68
**17**	1.38	0.10	4.35****	1.19	1.59
**18**	1.30	0.10	3.21***	1.11	1.52
**19**	1.38	0.11	4.11****	1.18	1.61
**20**	1.40	0.12	3.83****	1.18	1.66
**21**	1.35	0.12	3.31***	1.13	1.62
**22**	1.33	0.11	3.62****	1.14	1.56
**23**	1.26	0.11	2.61***	1.06	1.49
**24**	1.40	0.12	3.9****	1.18	1.65
**cons**	0.01	0.00	-82.58****	0.01	0.01
Chi-square = 215.92****					

Exhibit A30: Relationships among VDC receipt and changes in ambulatory care sensitive - hospital use over time (Fixed Effects Logit Model, matched VCD and comparison groups across all sites; N = 97,171 person-months)

Variable	OR	SE	Z	95% CI	
VD	1.15	0.19	0.83	0.83	1.58
NO_OFFER_POST	1.17	0.06	3.29***	1.07	1.29
**month**					
**2**	1.08	0.10	0.84	0.90	1.29
**3**	1.14	0.10	1.45	0.96	1.36
**4**	1.24	0.11	2.43**	1.04	1.47
**5**	1.39	0.12	3.85****	1.18	1.65
**6**	1.34	0.12	3.33***	1.13	1.58
**7**	1.36	0.12	3.53****	1.15	1.61
**8**	1.34	0.12	3.38***	1.13	1.59
**9**	1.52	0.13	4.96****	1.29	1.80
**10**	1.75	0.15	6.76****	1.49	2.06
**11**	1.99	0.16	8.46****	1.70	2.33
**12**	2.31	0.18	10.5****	1.98	2.70
**13**	1.22	0.11	2.19**	1.02	1.45
**14**	1.44	0.13	4.14****	1.21	1.71
**15**	1.61	0.14	5.48****	1.36	1.91
**16**	1.56	0.14	5.05****	1.31	1.86
**17**	1.49	0.13	4.41****	1.25	1.77
**18**	1.34	0.12	3.13***	1.11	1.60
**19**	1.49	0.14	4.34****	1.24	1.78
**20**	1.46	0.14	4.13****	1.22	1.76
**21**	1.49	0.14	4.3****	1.24	1.79
**22**	1.40	0.13	3.58****	1.17	1.69
**23**	1.30	0.13	2.75***	1.08	1.58
**24**	1.55	0.15	4.68****	1.29	1.87

Chi-square = 275.21****

Exhibit A31: Relationships among VDC receipt and changes in ambulatory care sensitive - hospital use over time (Population-Averaged Logit Model, matched across the entire sample at all sites; N = 843,540 person-months)

Variable	OR	SE	Z	95% CI	
VD	1.05	0.13	0.38	0.83	1.33
NO_OFFER_POST	1.22	0.05	5.29****	1.13	1.32
**month**					
**2**	1.07	0.09	0.84	0.91	1.27
**3**	1.13	0.09	1.46	0.96	1.33
**4**	1.22	0.10	2.44**	1.04	1.43
**5**	1.36	0.11	3.85****	1.16	1.59
**6**	1.31	0.11	3.34***	1.12	1.53
**7**	1.33	0.11	3.54****	1.13	1.55
**8**	1.31	0.11	3.39***	1.12	1.54
**9**	1.47	0.12	4.96****	1.26	1.72
**10**	1.67	0.13	6.73****	1.44	1.95
**11**	1.88	0.14	8.4****	1.62	2.18
**12**	2.14	0.16	10.38****	1.86	2.48
**13**	1.18	0.10	2**	1.00	1.39
**14**	1.36	0.11	3.85****	1.16	1.60
**15**	1.50	0.12	5.12****	1.29	1.75
**16**	1.44	0.12	4.56****	1.23	1.69
**17**	1.37	0.11	3.83****	1.16	1.60
**18**	1.23	0.10	2.47**	1.04	1.45
**19**	1.34	0.11	3.57****	1.14	1.58
**20**	1.32	0.11	3.32***	1.12	1.55
**21**	1.33	0.11	3.42***	1.13	1.57
**22**	1.25	0.11	2.67***	1.06	1.48
**23**	1.17	0.10	1.82*	0.99	1.39
**24**	1.36	0.11	3.66****	1.15	1.60
**cons**	0.01	0.00	-79.12****	0.01	0.01
Chi-square = 284.48****					

Exhibit A32: Relationships among VDC receipt and changes in ambulatory care sensitive - hospital use over time (Population-Averaged Logit Model, unmatched at active sites; N = 500,802 person-months)					
**Variable**	**OR**	**SE**	**Z**	**95% CI**	
VD	1.04	0.14	0.26	0.79	1.36
**month**					
**2**	1.11	0.12	0.97	0.90	1.36
**3**	1.22	0.16	1.53	0.95	1.56
**4**	1.36	0.16	2.73***	1.09	1.71
**5**	1.49	0.16	3.6***	1.20	1.85
**6**	1.43	0.16	3.19***	1.15	1.79
**7**	1.40	0.13	3.66****	1.17	1.68
**8**	1.37	0.15	2.81***	1.10	1.71
**9**	1.49	0.17	3.48****	1.19	1.86
**10**	1.74	0.21	4.57****	1.37	2.20
**11**	2.04	0.21	6.84****	1.67	2.51
**12**	2.22	0.21	8.65****	1.86	2.67
**13**	1.35	0.13	3.13***	1.12	1.62
**14**	1.57	0.15	4.77****	1.30	1.89
**15**	1.78	0.17	5.93****	1.47	2.16
**16**	1.53	0.16	3.91****	1.24	1.89
**17**	1.54	0.17	4.02****	1.25	1.90
**18**	1.57	0.20	3.54****	1.22	2.01
**19**	1.50	0.16	3.94****	1.23	1.84
**20**	1.61	0.18	4.37****	1.30	2.00
**21**	1.37	0.16	2.75***	1.09	1.71
**22**	1.37	0.18	2.39**	1.06	1.77
**23**	1.15	0.13	1.2	0.92	1.44
**24**	1.45	0.16	3.41***	1.17	1.79
**cons**	0.01	0.00	-62.71****	0.01	0.01
Chi-square = 329.48****					

Exhibit A33: Relationships among VDC receipt and changes in ambulatory care sensitive - hospital use over time (Fixed Effects Logit Model, matched among VDC group and comparison group at active sites; N = 50,423 person-months)

Variable	OR	SE	Z	95% CI	
VD	1.15	0.19	0.86	0.83	1.59
**month**					
**2**	1.07	0.13	0.52	0.84	1.36
**3**	1.16	0.14	1.26	0.92	1.48
**4**	1.34	0.16	2.48**	1.06	1.69
**5**	1.59	0.18	4.09****	1.28	1.99
**6**	1.44	0.17	3.12***	1.14	1.80
**7**	1.49	0.17	3.43***	1.19	1.86
**8**	1.46	0.17	3.27***	1.16	1.83
**9**	1.62	0.18	4.22****	1.29	2.02
**10**	2.07	0.23	6.6****	1.67	2.56
**11**	2.12	0.23	6.87****	1.71	2.63
**12**	2.45	0.26	8.36****	1.99	3.03
**13**	1.24	0.15	1.81*	0.98	1.57
**14**	1.62	0.19	4.22****	1.30	2.03
**15**	1.94	0.22	5.87****	1.55	2.41
**16**	1.67	0.19	4.4****	1.33	2.10
**17**	1.65	0.19	4.25****	1.31	2.08
**18**	1.58	0.19	3.81****	1.25	1.99
**19**	1.47	0.18	3.14***	1.16	1.87
**20**	1.67	0.20	4.26****	1.32	2.11
**21**	1.49	0.19	3.23***	1.17	1.91
**22**	1.38	0.18	2.52**	1.07	1.77
**23**	1.14	0.15	1	0.88	1.49
**24**	1.56	0.20	3.55****	1.22	2.00
Chi-square = 191.74 ****					

Exhibit A34: Relationships among VDC receipt and changes in ambulatory care sensitive - hospital use over time (Population-Averaged Logit Model, matched among VDC and comparison group at active sites; N = 497,675 person-months)

**Variable**	**OR**	**SE**	**Z**	**95% CI**	
VD	1.04	0.13	0.34	0.82	1.32
**month**					
**2**	1.06	0.12	0.52	0.85	1.33
**3**	1.15	0.13	1.27	0.93	1.43
**4**	1.31	0.14	2.48**	1.06	1.62
**5**	1.53	0.16	4.08****	1.25	1.88
**6**	1.40	0.15	3.12***	1.13	1.72
**7**	1.44	0.15	3.43***	1.17	1.77
**8**	1.42	0.15	3.27***	1.15	1.75
**9**	1.55	0.16	4.21****	1.27	1.91
**10**	1.93	0.19	6.54****	1.59	2.35
**11**	1.98	0.20	6.8****	1.63	2.41
**12**	2.25	0.22	8.23****	1.85	2.73
**13**	1.23	0.13	1.85*	0.99	1.52
**14**	1.55	0.16	4.16****	1.26	1.91
**15**	1.80	0.19	5.71****	1.47	2.20
**16**	1.56	0.17	4.2****	1.27	1.92
**17**	1.53	0.16	3.97****	1.24	1.89
**18**	1.46	0.16	3.46***	1.18	1.80
**19**	1.35	0.15	2.75***	1.09	1.68
**20**	1.51	0.16	3.79****	1.22	1.87
**21**	1.36	0.15	2.74***	1.09	1.69
**22**	1.26	0.14	2.03**	1.01	1.58
**23**	1.07	0.13	0.55	0.84	1.35
**24**	1.40	0.16	2.99***	1.12	1.74
**cons**	0.01	0.00	-59.43****	0.01	0.01

Chi-square = 195.42 ****

Exhibit A35: Relationships among VDC receipt and changes in ambulatory care sensitive - hospital use over time (Fixed Effects Logit Model, unmatched among VDC group and comparison group at active sites; N = 50,776 person-months)

Variable	OR	SE	Z	95% CI	
VD	1.09	0.21	0.43	0.75	1.57
**month**					
**2**	1.12	0.11	1.17	0.93	1.35
**3**	1.24	0.13	2**	1.00	1.52
**4**	1.40	0.17	2.85***	1.11	1.77
**5**	1.54	0.15	4.36****	1.27	1.88
**6**	1.48	0.17	3.52****	1.19	1.85
**7**	1.45	0.17	3.15***	1.15	1.82
**8**	1.41	0.15	3.15***	1.14	1.75
**9**	1.54	0.15	4.48****	1.28	1.87
**10**	1.84	0.20	5.6****	1.48	2.27
**11**	2.20	0.22	7.77****	1.81	2.69
**12**	2.43	0.19	11.62****	2.09	2.82
**13**	1.38	0.16	2.72***	1.09	1.74
**14**	1.65	0.21	3.95****	1.29	2.12
**15**	1.93	0.25	5.14****	1.50	2.47
**16**	1.64	0.19	4.34****	1.31	2.04
**17**	1.68	0.19	4.56****	1.34	2.10
**18**	1.73	0.19	5.09****	1.40	2.13
**19**	1.67	0.18	4.9****	1.36	2.05
**20**	1.82	0.19	5.6****	1.48	2.25
**21**	1.53	0.15	4.17****	1.25	1.86
**22**	1.53	0.20	3.33***	1.19	1.97
**23**	1.26	0.14	2.08**	1.01	1.57
**24**	1.65	0.21	4****	1.29	2.12

Chi-square = 450.81 ****

Exhibit A36: Relationships among VDC receipt and changes in ambulatory care sensitive - hospital use over time; sensitivity analysis of a different definition of post period (Fixed Effects Logit Model, matched among entire sample; N = 97,171 person-months)					
**Variable**	**OR**	**SE**	**Z**	**95% CI**	
VD	1.12	0.19	0.66	0.80	1.57
NO_OFFER_POST	1.15	0.06	2.79***	1.04	1.27
**month**					
**2**	1.08	0.10	0.84	0.90	1.29
**3**	1.14	0.10	1.45	0.96	1.36
**4**	1.24	0.11	2.43**	1.04	1.47
**5**	1.39	0.12	3.85****	1.18	1.65
**6**	1.34	0.12	3.33***	1.13	1.58
**7**	1.36	0.12	3.53****	1.15	1.61
**8**	1.34	0.12	3.38***	1.13	1.59
**9**	1.52	0.13	4.96****	1.29	1.80
**10**	1.75	0.15	6.76****	1.49	2.06
**11**	1.99	0.16	8.46****	1.70	2.33
**12**	2.31	0.18	10.5****	1.98	2.70
**13**	1.31	0.11	3.14***	1.11	1.56
**14**	1.55	0.13	5.18****	1.32	1.84
**15**	1.63	0.14	5.57****	1.37	1.93
**16**	1.58	0.14	5.14****	1.33	1.88
**17**	1.50	0.14	4.5****	1.26	1.79
**18**	1.35	0.13	3.23***	1.12	1.62
**19**	1.50	0.14	4.43****	1.25	1.80
**20**	1.48	0.14	4.22****	1.23	1.77
**21**	1.50	0.14	4.39****	1.25	1.81
**22**	1.42	0.13	3.67****	1.18	1.71
**23**	1.32	0.13	2.83***	1.09	1.59
**24**	1.57	0.15	4.77****	1.30	1.89
Chi-square = 272.06 ****					

Exhibit A37: Relationships among VDC receipt and changes in ambulatory care sensitive - hospital use over time; sensitivity analysis of patients with GEC services (Fixed Effects Logit Model, matched among entire sample; N = 95,478 person-months)

Variable	OR	SE	Z	95% CI	
VD	1.18	0.25	0.77	0.78	1.78
NO_OFFER_POST	1.13	0.05	2.68***	1.03	1.25
**month**					
**2**	1.11	0.10	1.22	0.94	1.32
**3**	1.14	0.10	1.47	0.96	1.35
**4**	1.22	0.10	2.37**	1.04	1.45
**5**	1.34	0.11	3.46***	1.13	1.58
**6**	1.29	0.11	3.03***	1.09	1.52
**7**	1.30	0.11	3.12***	1.10	1.53
**8**	1.33	0.11	3.43***	1.13	1.57
**9**	1.49	0.12	4.84****	1.27	1.75
**10**	1.77	0.14	7.16****	1.52	2.07
**11**	1.92	0.15	8.26****	1.64	2.24
**12**	2.33	0.18	10.98****	2.00	2.71
**13**	1.19	0.10	2.03**	1.01	1.42
**14**	1.40	0.12	3.9****	1.18	1.65
**15**	1.58	0.13	5.41****	1.34	1.87
**16**	1.54	0.13	5.03****	1.30	1.82
**17**	1.47	0.13	4.42****	1.24	1.75
**18**	1.35	0.12	3.36***	1.13	1.61
**19**	1.46	0.13	4.26****	1.23	1.74
**20**	1.42	0.13	3.88****	1.19	1.69
**21**	1.50	0.13	4.52****	1.26	1.79
**22**	1.40	0.13	3.63****	1.17	1.67
**23**	1.29	0.12	2.71***	1.07	1.55
**24**	1.53	0.14	4.68****	1.28	1.83
Chi-square = 283.15 ****					

Exhibit A38: Relationships among VDC receipt and changes in ambulatory care sensitive - hospital use over time; sensitivity analysis of patients at least 3 months of service duration (Fixed Effects Logit Model, matched among entire sample; N = 82,427 person-months)

Variable	OR	SE	Z	95% CI	
VD	1.17	0.20	0.91	0.83	1.64
NO_OFFER_POST	1.12	0.06	2.15**	1.01	1.24
**month**					
**2**	1.06	0.11	0.61	0.87	1.29
**3**	1.20	0.12	1.91*	0.99	1.45
**4**	1.21	0.12	1.96*	1.00	1.46
**5**	1.40	0.13	3.54****	1.16	1.68
**6**	1.28	0.12	2.55**	1.06	1.54
**7**	1.22	0.12	2.06**	1.01	1.47
**8**	1.24	0.12	2.25**	1.03	1.50
**9**	1.48	0.14	4.23****	1.24	1.78
**10**	1.73	0.16	6.07****	1.45	2.07
**11**	1.82	0.16	6.64****	1.52	2.17
**12**	2.04	0.18	8.06****	1.72	2.43
**13**	0.94	0.10	-0.6	0.77	1.15
**14**	1.17	0.12	1.57	0.96	1.42
**15**	1.55	0.15	4.64****	1.29	1.87
**16**	1.51	0.14	4.27****	1.25	1.82
**17**	1.42	0.14	3.62****	1.18	1.72
**18**	1.33	0.13	2.84***	1.09	1.61
**19**	1.46	0.14	3.83****	1.20	1.77
**20**	1.44	0.14	3.65****	1.18	1.74
**21**	1.35	0.14	3.01***	1.11	1.65
**22**	1.36	0.14	2.99***	1.11	1.66
**23**	1.29	0.13	2.45**	1.05	1.58
**24**	1.56	0.16	4.46****	1.29	1.91

Chi-square = 202.46****

Exhibit A39: Relationships among VDC receipt and changes in ambulatory care sensitive - hospital use over time; sensitivity analysis of patients at least 6 months of service duration (Fixed Effects Logit Model, matched among entire sample; N = 66,818 person-months)

Variable	OR	SE	Z	95% CI	
VD	1.07	0.20	0.38	0.74	1.56
NO_OFFER_POST	1.21	0.07	3.31***	1.08	1.36
**month**					
**2**	1.06	0.12	0.53	0.85	1.31
**3**	1.16	0.13	1.41	0.94	1.44
**4**	1.07	0.12	0.64	0.87	1.33
**5**	1.38	0.14	3.09***	1.13	1.69
**6**	1.24	0.13	2.05**	1.01	1.53
**7**	1.16	0.13	1.4	0.94	1.44
**8**	1.15	0.12	1.3	0.93	1.42
**9**	1.40	0.15	3.24***	1.14	1.72
**10**	1.72	0.17	5.4****	1.41	2.10
**11**	1.80	0.18	5.87****	1.48	2.19
**12**	1.98	0.19	6.91****	1.63	2.40
**13**	0.83	0.10	-1.61	0.66	1.04
**14**	1.01	0.11	0.07	0.81	1.26
**15**	1.30	0.14	2.45**	1.05	1.61
**16**	1.14	0.13	1.16	0.92	1.41
**17**	1.16	0.13	1.36	0.94	1.44
**18**	1.14	0.13	1.21	0.92	1.42
**19**	1.36	0.15	2.89***	1.11	1.68
**20**	1.31	0.14	2.47**	1.06	1.62
**21**	1.25	0.14	2.02**	1.01	1.55
**22**	1.16	0.13	1.36	0.93	1.45
**23**	1.12	0.13	0.99	0.90	1.40
**24**	1.41	0.15	3.12***	1.14	1.74
Chi-square = 176.86****					

Exhibit A40: Relationships among VDC receipt and changes in acute care non-zero costs over time, entire sample, unmatched (Fixed Effects Linear Regression Model; N = 307,955 person-months)					
**Variable**	**Coef.**	**SE**	**Z**	**95% CI**	
**VD**	-1089.57	550.74	-1.98**	-2169.01	-10.13
**NO_OFFER_POST**	201.23	145.55	1.38	-84.03	486.50
**month**					
**2**	90.73	94.11	0.96	-93.73	275.19
**3**	83.37	116.37	0.72	-144.71	311.45
**4**	282.41	118.29	2.39**	50.57	514.25
**5**	421.63	136.38	3.09***	154.33	688.93
**6**	505.88	119.12	4.25****	272.41	739.34
**7**	757.80	131.09	5.78****	500.86	1014.74
**8**	737.96	104.46	7.06****	533.22	942.70
**9**	1304.77	117.98	11.06****	1073.54	1536.00
**10**	1891.94	125.79	15.04****	1645.40	2138.48
**11**	2292.12	129.81	17.66****	2037.69	2546.54
**12**	1694.62	117.49	14.42****	1464.34	1924.91
**13**	182.70	140.37	1.3	-92.43	457.82
**14**	741.33	147.52	5.03****	452.20	1030.45
**15**	808.56	153.13	5.28****	508.43	1108.68
**16**	1006.73	156.80	6.42****	699.40	1314.05
**17**	1118.57	181.40	6.17****	763.03	1474.10
**18**	1124.80	158.83	7.08****	813.51	1436.10
**19**	1060.23	162.56	6.52****	741.62	1378.83
**20**	1241.66	155.84	7.97****	936.22	1547.10
**21**	1099.75	168.90	6.51****	768.71	1430.79
**22**	1209.47	163.69	7.39****	888.65	1530.29
**23**	960.63	137.25	7****	691.62	1229.65
**24**	1030.41	158.93	6.48****	718.91	1341.92
**_cons**	2039.39	84.12	24.24****	1874.51	2204.27
Chi-square = 1453.29****					

Exhibit A41: Relationships among VDC receipt and changes in acute care non-zero costs over time, entire sample, unmatched (Population-Averaged GLM with gamma family, log link; N = 307,955 person-months)

Variable	Average Incremental Effect	Std. Err.	z	95% C. I.	
**VD**	-348.27	260.45	-1.34	-858.73	162.20
**NO_OFFER_POST**	-35.98	112.47	-0.32	-256.42	184.46
	**Coef.**	**SE**	**Z**	**95% CI**	
**VD**	-0.12	0.10	-1.26	-0.31	0.07
**NO_OFFER_POST**	-0.01	0.04	-0.32	-0.09	0.06
**month**					
**2**	0.04	0.04	0.96	-0.04	0.13
**3**	0.04	0.05	0.71	-0.07	0.14
**4**	0.12	0.05	2.39**	0.02	0.23
**5**	0.18	0.06	3.12***	0.07	0.30
**6**	0.21	0.05	4.29****	0.12	0.31
**7**	0.31	0.05	5.99****	0.21	0.41
**8**	0.30	0.04	6.8****	0.21	0.38
**9**	0.48	0.04	10.79****	0.39	0.57
**10**	0.64	0.05	14.03****	0.55	0.73
**11**	0.73	0.04	17.34****	0.65	0.82
**12**	0.59	0.04	13.17****	0.50	0.67
**13**	0.12	0.05	2.21**	0.01	0.22
**14**	0.33	0.05	6.13****	0.22	0.43
**15**	0.35	0.05	6.35****	0.24	0.45
**16**	0.41	0.06	7.42****	0.30	0.52
**17**	0.44	0.06	7.02****	0.32	0.56
**18**	0.44	0.06	7.94****	0.33	0.55
**19**	0.41	0.06	7.33****	0.30	0.52
**20**	0.47	0.05	8.75****	0.37	0.58
**21**	0.42	0.06	7.37****	0.31	0.54
**22**	0.46	0.06	8.05****	0.35	0.57
**23**	0.37	0.05	7.06****	0.27	0.47
**24**	0.39	0.06	6.81****	0.28	0.51
**_cons**	7.66	0.04	194.83****	7.58	7.74

Chi-square = 1537.89****

Exhibit A42: Relationships among VDC receipt and changes in hospital use over time (Fixed Effects Logit Model, matched VCD and comparison groups across all sites; at least 3 months of service duration; N = 258,161 person-months)

Variable	OR	SE	Z	95% CI	
VD	0.90	0.07	-1.38	0.77	1.04
NO_OFFER_POST	1.06	0.03	2.34**	1.01	1.11
**month**					
**2**	1.04	0.04	0.82	0.95	1.13
**3**	1.06	0.05	1.4	0.98	1.15
**4**	1.13	0.05	2.89****	1.04	1.23
**5**	1.19	0.05	4.09****	1.09	1.29
**6**	1.16	0.05	3.54****	1.07	1.26
**7**	1.20	0.05	4.34****	1.10	1.30
**8**	1.30	0.05	6.34****	1.20	1.41
**9**	1.40	0.06	8.15****	1.29	1.51
**10**	1.66	0.07	12.69****	1.54	1.80
**11**	1.81	0.07	15.02****	1.68	1.96
**12**	1.68	0.07	13.03****	1.56	1.82
**13**	0.78	0.04	-5.35****	0.71	0.86
**14**	0.93	0.04	-1.72*	0.85	1.01
**15**	1.15	0.05	3.25***	1.06	1.25
**16**	1.25	0.05	5.1****	1.14	1.36
**17**	1.25	0.05	5.06****	1.14	1.36
**18**	1.28	0.06	5.63****	1.17	1.39
**19**	1.33	0.06	6.53****	1.22	1.45
**20**	1.32	0.06	6.39****	1.21	1.44
**21**	1.27	0.06	5.44****	1.17	1.39
**22**	1.32	0.06	6.21****	1.21	1.43
**23**	1.21	0.05	4.19****	1.11	1.32
**24**	1.30	0.06	5.88****	1.19	1.42

Chi-square = 943.42****

Exhibit A43: Relationships among VDC receipt and changes in hospital use over time (Fixed Effects Logit Model, matched VCD and comparison groups across all sites; at least 6 months of service duration; N = 214,188 person-months)

Variable	OR	SE	Z	95% CI	
VD	0.91	0.07	-1.16	0.78	1.07
NO_OFFER_POST	1.08	0.03	2.93***	1.03	1.14
**month**					
**2**	1.03	0.05	0.6	0.94	1.13
**3**	1.05	0.05	0.99	0.96	1.15
**4**	1.11	0.05	2.22***	1.01	1.21
**5**	1.17	0.05	3.4***	1.07	1.28
**6**	1.15	0.05	2.93***	1.05	1.25
**7**	1.17	0.05	3.47****	1.07	1.28
**8**	1.27	0.06	5.17****	1.16	1.38
**9**	1.33	0.06	6.36****	1.22	1.46
**10**	1.58	0.07	10.43****	1.45	1.73
**11**	1.71	0.07	12.23****	1.57	1.86
**12**	1.52	0.07	9.51****	1.40	1.66
**13**	0.71	0.04	-6.56****	0.64	0.79
**14**	0.83	0.04	-3.76****	0.75	0.91
**15**	0.95	0.05	-0.99	0.87	1.05
**16**	0.97	0.05	-0.53	0.89	1.07
**17**	0.97	0.05	-0.63	0.88	1.07
**18**	1.07	0.05	1.39	0.97	1.17
**19**	1.18	0.06	3.47***	1.07	1.29
**20**	1.18	0.06	3.49****	1.08	1.30
**21**	1.16	0.06	3.07***	1.05	1.27
**22**	1.21	0.06	4.05****	1.11	1.33
**23**	1.14	0.06	2.62***	1.03	1.25
**24**	1.22	0.06	4.18****	1.11	1.35
Chi-square = 783.79****					

Exhibit A44: Relationships among VDC receipt and changes in acute care costs over time (Fixed Effects Linear Regression Model; matched VDC and comparison groups across all sites; at least 3 months of service duration; denominator: entire sample; N = 733,440 person-months)

Variable	Coef.	SE	t	95% CI	
**VD**	-215.23	100.06	-2.15**	-411.35	-19.11
**NO_OFFER_POST**	73.84	32.98	2.24**	9.21	138.47
**month**					
**2**	61.30	54.54	1.12	-45.60	168.20
**3**	11.16	54.54	0.2	-95.74	118.06
**4**	86.93	54.54	1.59	-19.97	193.82
**5**	144.10	54.54	2.64***	37.20	251.00
**6**	164.24	54.54	3.01***	57.34	271.13
**7**	206.38	54.54	3.78****	99.49	313.28
**8**	249.63	54.54	4.58****	142.74	356.53
**9**	429.09	54.54	7.87****	322.19	535.99
**10**	686.28	54.54	12.58****	579.39	793.18
**11**	633.19	54.54	11.61****	526.29	740.09
**12**	353.59	54.54	6.48****	246.69	460.49
**13**	-362.14	56.36	-6.43****	-472.60	-251.68
**14**	-252.25	56.36	-4.48****	-362.72	-141.79
**15**	-67.99	56.36	-1.21	-178.46	42.47
**16**	121.97	56.53	2.16**	11.17	232.78
**17**	176.20	56.77	3.1***	64.93	287.46
**18**	227.65	57.02	3.99****	115.89	339.41
**19**	267.22	57.27	4.67****	154.97	379.47
**20**	263.87	57.52	4.59****	151.13	376.60
**21**	251.66	57.79	4.35****	138.40	364.93
**22**	277.93	58.03	4.79****	164.19	391.67
**23**	159.11	58.28	2.73***	44.88	273.34
**24**	250.83	58.53	4.29****	136.11	365.55
**_cons**	993.31	38.57	25.75****	917.71	1068.92
F = 32.28****					

Exhibit A45: Relationships among VDC receipt and changes in acute care costs over time (Population-averaged GLM Model with gamma distribution; at least 3 months of service duration; matched; denominator: entire sample; N = 733,440 person-months)

Variable	Average Incremental Effect	Std. Err.	z	95% C. I.	
**VD**	-63.68	89.11	-0.71	-238.33	110.97
**NO_OFFER_POST**	39.40	31.60	1.25	-22.53	101.32
	**Coef.**	**SE**	**Z**	**95% CI**	
**VD**	-0.05	0.08	-0.7	-0.21	0.10
**NO_OFFER_POST**	0.03	0.03	1.26	-0.02	0.08
**month**					
**2**	0.06	0.05	1.28	-0.03	0.15
**3**	0.01	0.05	0.24	-0.08	0.10
**4**	0.08	0.05	1.79*	-0.01	0.17
**5**	0.13	0.05	2.89***	0.04	0.22
**6**	0.15	0.05	3.27***	0.06	0.24
**7**	0.19	0.05	4.03****	0.10	0.28
**8**	0.22	0.05	4.79****	0.13	0.31
**9**	0.35	0.05	7.68****	0.26	0.44
**10**	0.52	0.05	11.24****	0.43	0.61
**11**	0.49	0.05	10.55****	0.40	0.58
**12**	0.30	0.05	6.51****	0.21	0.39
**13**	-0.42	0.05	-8.83****	-0.51	-0.33
**14**	-0.27	0.05	-5.64****	-0.36	-0.17
**15**	-0.05	0.05	-1.15	-0.15	0.04
**16**	0.12	0.05	2.63***	0.03	0.22
**17**	0.17	0.05	3.52****	0.07	0.26
**18**	0.21	0.05	4.35****	0.11	0.30
**19**	0.24	0.05	4.96****	0.14	0.33
**20**	0.24	0.05	4.9****	0.14	0.33
**21**	0.23	0.05	4.64****	0.13	0.32
**22**	0.25	0.05	5.01****	0.15	0.34
**23**	0.14	0.05	2.92***	0.05	0.24
**24**	0.22	0.05	4.5****	0.13	0.32
**_cons**	6.92	0.04	193.61****	6.85	6.99

Chi-square = 879.85****

Exhibit A46: Relationships among VDC receipt and changes in acute care costs over time (Fixed Effects Linear Regression Model; matched VDC and comparison groups across all sites; at least 6 months of service duration; denominator: entire sample; N 628,175 person-months)

Variable	Coef.	SE	t	95% CI	
**VD**	-261.81	100.53	-2.6***	-458.85	-64.78
**NO_OFFER_POST**	95.94	33.94	2.83***	29.42	162.46
**month**					
**2**	63.80	56.84	1.12	-47.59	175.20
**3**	-23.71	56.84	-0.42	-135.11	87.69
**4**	61.31	56.84	1.08	-50.09	172.70
**5**	129.19	56.84	2.27**	17.80	240.59
**6**	115.48	56.84	2.03**	4.08	226.87
**7**	166.38	56.84	2.93***	54.98	277.78
**8**	219.34	56.84	3.86****	107.94	330.74
**9**	370.05	56.84	6.51****	258.65	481.44
**10**	616.57	56.84	10.85****	505.18	727.97
**11**	538.50	56.84	9.47****	427.10	649.89
**12**	228.21	56.84	4.02****	116.81	339.61
**13**	-429.82	58.67	-7.33****	-544.82	-314.82
**14**	-351.63	58.67	-5.99****	-466.62	-236.63
**15**	-280.69	58.67	-4.78****	-395.69	-165.69
**16**	-204.50	58.67	-3.49****	-319.49	-89.50
**17**	-140.29	58.67	-2.39**	-255.28	-25.29
**18**	-37.04	58.67	-0.63	-152.04	77.96
**19**	33.95	58.80	0.58	-81.29	149.20
**20**	107.24	58.98	1.82*	-8.36	222.85
**21**	156.49	59.22	2.64***	40.43	272.56
**22**	153.62	59.43	2.58**	37.14	270.10
**23**	62.96	59.66	1.06	-53.98	179.90
**24**	159.57	59.89	2.66***	42.18	276.96
**_cons**	1001.15	40.19	24.91****	922.37	1079.92

F = 32.68****

Exhibit A47: Relationships among VDC receipt and changes in acute care costs over time (Population-averaged GLM Model with gamma distribution; at least 6 months of service duration; matched; denominator: entire sample; N = 628,175 person-months)

Variable	Average Incremental Effect	Std. Err.	z	95% C. I.	
VD	-42.14	91.00	-0.46	-220.49	136.21
NO_OFFER_POST	34.74	32.57	1.07	-29.10	98.58
	**Coef.**	**SE**	**Z**	**95% CI**	
VD	-0.04	0.09	-0.45	-0.21	0.13
NO_OFFER_POST	0.03	0.03	1.08	-0.03	0.09
month					
2	0.06	0.05	1.16	-0.04	0.17
3	-0.02	0.05	-0.45	-0.13	0.08
4	0.06	0.05	1.12	-0.04	0.16
5	0.12	0.05	2.28**	0.02	0.23
6	0.11	0.05	2.05**	0.00	0.21
7	0.15	0.05	2.89***	0.05	0.26
8	0.20	0.05	3.73****	0.09	0.30
9	0.31	0.05	5.92****	0.21	0.42
10	0.48	0.05	9.03****	0.37	0.58
11	0.43	0.05	8.1****	0.33	0.53
12	0.20	0.05	3.86****	0.10	0.31
13	-0.52	0.05	-9.5****	-0.63	-0.41
14	-0.40	0.05	-7.26****	-0.50	-0.29
15	-0.29	0.05	-5.4****	-0.40	-0.19
16	-0.20	0.05	-3.63****	-0.30	-0.09
17	-0.12	0.05	-2.29**	-0.23	-0.02
18	-0.02	0.05	-0.3	-0.12	0.09
19	0.05	0.05	0.97	-0.05	0.16
20	0.12	0.05	2.18**	0.01	0.23
21	0.16	0.06	2.94***	0.05	0.27
22	0.16	0.06	2.86***	0.05	0.27
23	0.08	0.06	1.38	-0.03	0.19
24	0.16	0.06	2.92***	0.05	0.27
_cons	6.91	0.04	168.36****	6.83	6.99

Chi-square = 863.44****

Exhibit A48: Relationships among VDC receipt and changes in acute care costs over time (Fixed Effects Linear Regression Model; matched VDC and comparison groups across all sites; non-decedents; denominator: entire sample; N = 696,336 person-months)

Variable	Coef.	SE	t	95% CI	
**VD**	-300.35	104.46	-2.88***	-505.09	-95.61
**NO_OFFER_POST**	113.65	33.70	3.37***	47.61	179.69
**month**					
**2**	30.42	56.79	0.54	-80.89	141.73
**3**	-7.24	56.79	-0.13	-118.55	104.07
**4**	55.57	56.79	0.98	-55.74	166.88
**5**	105.89	56.79	1.86*	-5.42	217.20
**6**	143.87	56.79	2.53**	32.56	255.18
**7**	260.36	56.79	4.58****	149.05	371.67
**8**	315.86	56.79	5.56****	204.55	427.17
**9**	510.09	56.79	8.98****	398.78	621.40
**10**	789.75	56.79	13.91****	678.44	901.06
**11**	718.30	56.79	12.65****	606.99	829.61
**12**	430.87	56.79	7.59****	319.56	542.18
**13**	-316.96	58.56	-5.41****	-431.73	-202.19
**14**	-147.20	58.56	-2.51**	-261.98	-32.43
**15**	-92.41	58.56	-1.58	-207.18	22.37
**16**	5.31	58.56	0.09	-109.46	120.08
**17**	28.62	58.56	0.49	-86.15	143.40
**18**	46.22	58.56	0.79	-68.55	161.00
**19**	69.21	58.56	1.18	-45.57	183.98
**20**	54.68	58.56	0.93	-60.09	169.45
**21**	66.72	58.56	1.14	-48.05	181.50
**22**	76.51	58.56	1.31	-38.26	191.28
**23**	27.55	58.56	0.47	-87.22	142.32
**24**	116.09	58.56	1.98**	1.31	230.86
**_cons**	1026.42	40.16	25.56****	947.71	1105.13

F = 35.19****

Exhibit A49: Relationships among VDC receipt and changes in acute care costs over time (Population-averaged GLM Model with gamma distribution; non-decedents; matched; denominator: entire sample; N 696,336 person-months)

Variable	Average Incremental Effect	Std. Err.	z	95% C. I.	
VD	-212.56	81.75	-2.6**	-372.78	-52.35
NO_OFFER_POST	36.90	32.59	1.13	-26.98	100.77
	**Coef.**	**SE**	**Z**	**95% CI**	
**VD**	-0.20	0.08	-2.36**	-0.36	-0.03
**NO_OFFER_POST**	0.03	0.03	1.14	-0.02	0.08
**month**					
**2**	0.03	0.05	0.6	-0.07	0.13
**3**	-0.01	0.05	-0.14	-0.10	0.09
**4**	0.05	0.05	1.08	-0.04	0.15
**5**	0.10	0.05	2.01**	0.00	0.19
**6**	0.13	0.05	2.68***	0.04	0.23
**7**	0.23	0.05	4.62****	0.13	0.32
**8**	0.27	0.05	5.48****	0.17	0.36
**9**	0.40	0.05	8.24****	0.31	0.50
**10**	0.57	0.05	11.66****	0.47	0.67
**11**	0.53	0.05	10.84****	0.43	0.63
**12**	0.35	0.05	7.16****	0.25	0.45
**13**	-0.33	0.05	-6.48****	-0.42	-0.23
**14**	-0.12	0.05	-2.42**	-0.22	-0.02
**15**	-0.06	0.05	-1.19	-0.16	0.04
**16**	0.04	0.05	0.75	-0.06	0.14
**17**	0.06	0.05	1.16	-0.04	0.16
**18**	0.07	0.05	1.47	-0.02	0.17
**19**	0.09	0.05	1.84*	-0.01	0.19
**20**	0.08	0.05	1.62	-0.02	0.18
**21**	0.09	0.05	1.81*	-0.01	0.19
**22**	0.10	0.05	1.96*	0.00	0.20
**23**	0.05	0.05	1.09	-0.04	0.15
**24**	0.13	0.05	2.68***	0.04	0.23
**_cons**	6.93	0.04	181.14****	6.86	7.01

Chi-square = 738.05****

Exhibit A50: Relationships among VDC receipt and changes in hospital use over time, with sample restricted to those alive at the end of the study period (Fixed Effects Model, unmatched sample; N = 248,280 person-months)

Variables	OR	SE	Z	95% CI	
VD	0.79	0.12	-1.52	0.59	1.07
NO_OFFER_POST	1.09	0.05	1.92*	1.00	1.19
**month**					
**2**	1.04	0.04	1.05	0.97	1.12
**3**	1.06	0.05	1.05	0.95	1.17
**4**	1.14	0.06	2.36**	1.02	1.27
**5**	1.14	0.06	2.41**	1.03	1.27
**6**	1.18	0.06	3.21***	1.07	1.30
**7**	1.27	0.07	4.33****	1.14	1.42
**8**	1.35	0.07	5.96****	1.22	1.49
**9**	1.44	0.08	6.44****	1.29	1.61
**10**	1.69	0.10	8.69****	1.50	1.91
**11**	2.02	0.10	13.55****	1.82	2.23
**12**	1.98	0.10	13.75****	1.80	2.19
**13**	0.92	0.06	-1.3	0.81	1.04
**14**	1.06	0.05	1.23	0.96	1.18
**15**	1.16	0.07	2.72***	1.04	1.30
**16**	1.15	0.06	2.63***	1.04	1.28
**17**	1.15	0.07	2.54**	1.03	1.29
**18**	1.15	0.06	2.71***	1.04	1.28
**19**	1.16	0.06	2.72***	1.04	1.29
**20**	1.17	0.07	2.48**	1.03	1.32
**21**	1.13	0.07	2.13**	1.01	1.27
**22**	1.19	0.07	3.11***	1.07	1.33
**23**	1.18	0.07	2.93***	1.06	1.32
**24**	1.27	0.07	4.3****	1.14	1.42
Chi-square = 1320.22****					

Exhibit A51: Relationships among VDC receipt and changes in hospital use over time, with sample restricted to those alive at the end of the study period (Fixed Effects Model, matched across entire sample; alive till end of study; N = 246,192 person-months)

Variables	OR	SE	Z	95% CI	
VD	0.81	0.07	-2.59**	0.68	0.95
NO_OFFER_POST	1.04	0.03	1.39	0.99	1.09
**month**					
**2**	1.02	0.05	0.45	0.93	1.11
**3**	1.04	0.05	0.82	0.95	1.13
**4**	1.13	0.05	2.68***	1.03	1.23
**5**	1.17	0.05	3.61****	1.08	1.28
**6**	1.13	0.05	2.79***	1.04	1.23
**7**	1.23	0.05	4.74****	1.13	1.34
**8**	1.35	0.06	6.87****	1.24	1.46
**9**	1.43	0.06	8.43****	1.32	1.56
**10**	1.70	0.07	12.67****	1.57	1.84
**11**	1.92	0.08	15.84****	1.77	2.08
**12**	1.84	0.08	14.76****	1.70	2.00
**13**	0.84	0.04	-3.61****	0.77	0.92
**14**	1.01	0.05	0.28	0.92	1.11
**15**	1.12	0.05	2.48**	1.02	1.22
**16**	1.17	0.05	3.42***	1.07	1.28
**17**	1.13	0.05	2.61***	1.03	1.23
**18**	1.11	0.05	2.24**	1.01	1.21
**19**	1.15	0.05	3***	1.05	1.25
**20**	1.14	0.05	2.79***	1.04	1.24
**21**	1.10	0.05	2.07**	1.00	1.20
**22**	1.14	0.05	2.88***	1.04	1.25
**23**	1.10	0.05	2.06**	1.00	1.20
**24**	1.21	0.05	4.14****	1.10	1.32

Chi-square = 1021.07****

Exhibit A52: Relationships among VDC receipt and changes in hospital use over time, with sample restricted to those alive at the end of the study period (Fixed Effects Model, matched across sample in active sites; alive until end of study; N = 135,192 person-months)

Variables	OR	SE	Z	95% CI	
VD	0.82	0.07	-2.36**	0.70	0.97
**month**					
**2**	1.02	0.06	0.26	0.90	1.14
**3**	1.03	0.06	0.56	0.92	1.16
**4**	1.18	0.07	2.93***	1.06	1.32
**5**	1.16	0.07	2.63***	1.04	1.30
**6**	1.13	0.07	2.18**	1.01	1.27
**7**	1.21	0.07	3.29***	1.08	1.35
**8**	1.37	0.08	5.66****	1.23	1.53
**9**	1.49	0.08	7.17****	1.34	1.66
**10**	1.69	0.09	9.58****	1.52	1.88
**11**	1.79	0.10	10.7****	1.61	1.99
**12**	1.93	0.10	12.17****	1.73	2.14
**13**	0.84	0.05	-2.79***	0.75	0.95
**14**	1.00	0.06	0.01	0.89	1.12
**15**	1.16	0.07	2.51**	1.03	1.29
**16**	1.13	0.07	2.06**	1.01	1.26
**17**	1.06	0.06	1	0.95	1.19
**18**	1.11	0.06	1.8*	0.99	1.24
**19**	1.11	0.06	1.77*	0.99	1.24
**20**	1.18	0.07	2.85***	1.05	1.32
**21**	1.08	0.06	1.2	0.96	1.21
**22**	1.14	0.07	2.31**	1.02	1.28
**23**	1.02	0.06	0.39	0.91	1.15
**24**	1.16	0.07	2.59**	1.04	1.30
Chi-square = 634.92****					

Exhibit A53: Relationships among VDC receipt and changes in hospital use over time, with sample restricted to those alive at the end of the study period (Population-averaged Model, unmatched; N = 700,776 person-months)

Variables	OR	SE	Z	95% CI	
VD	0.87	0.09	-1.35	0.71	1.07
**NO_OFFER_POST**	1.10	0.03	3.65****	1.04	1.15
**month**					
**2**	1.03	0.04	0.99	0.97	1.11
**3**	1.05	0.04	1.31	0.98	1.12
**4**	1.12	0.04	3.6****	1.05	1.19
**5**	1.12	0.04	2.93***	1.04	1.21
**6**	1.15	0.05	3.26***	1.06	1.26
**7**	1.23	0.05	5.09****	1.14	1.33
**8**	1.30	0.05	7.07****	1.21	1.39
**9**	1.37	0.05	8.89****	1.28	1.47
**10**	1.57	0.06	12.61****	1.46	1.68
**11**	1.81	0.06	17.38****	1.70	1.94
**12**	1.79	0.07	14.84****	1.66	1.93
**13**	0.92	0.04	-1.79*	0.84	1.01
**14**	1.05	0.05	0.94	0.95	1.15
**15**	1.13	0.05	2.55**	1.03	1.24
**16**	1.12	0.04	2.96***	1.04	1.21
**17**	1.12	0.05	2.49**	1.02	1.23
**18**	1.12	0.04	2.85***	1.04	1.21
**19**	1.13	0.05	2.64***	1.03	1.23
**20**	1.13	0.05	2.62***	1.03	1.24
**21**	1.10	0.04	2.48**	1.02	1.20
**22**	1.15	0.05	3.69****	1.07	1.25
**23**	1.14	0.04	3.43***	1.06	1.23
**24**	1.22	0.05	4.72***	1.12	1.32
**_cons**	0.04	0.00	-112.78****	0.03	0.04
Chi-square = 962.17****					

Exhibit A54: Relationships among VDC receipt and changes in hospital use over time, with sample restricted to those alive at the end of the study period (Population-averaged Model, matched entire sample; N = 696,336 person-months)

Variables	OR	SE	Z	95% CI	
VD	0.84	0.06	-2.53**	0.74	0.96
**NO_OFFER_POST**	1.03	0.02	1.58	0.99	1.07
**month**					
**2**	1.02	0.04	0.45	0.94	1.10
**3**	1.03	0.04	0.82	0.96	1.11
**4**	1.11	0.04	2.7***	1.03	1.19
**5**	1.14	0.04	3.62****	1.06	1.23
**6**	1.11	0.04	2.8***	1.03	1.19
**7**	1.19	0.04	4.76****	1.11	1.28
**8**	1.28	0.05	6.87****	1.19	1.38
**9**	1.35	0.05	8.41****	1.26	1.45
**10**	1.55	0.05	12.56****	1.45	1.66
**11**	1.71	0.06	15.64****	1.60	1.83
**12**	1.66	0.06	14.6****	1.55	1.77
**13**	0.86	0.03	-3.68****	0.80	0.93
**14**	1.01	0.04	0.26	0.94	1.09
**15**	1.10	0.04	2.48**	1.02	1.19
**16**	1.14	0.04	3.43***	1.06	1.23
**17**	1.11	0.04	2.62***	1.03	1.19
**18**	1.09	0.04	2.24**	1.01	1.17
**19**	1.12	0.04	3***	1.04	1.21
**20**	1.11	0.04	2.8***	1.03	1.20
**21**	1.08	0.04	2.07**	1.00	1.17
**22**	1.12	0.04	2.89***	1.04	1.20
**23**	1.08	0.04	2.06**	1.00	1.17
**24**	1.17	0.04	4.15****	1.09	1.26
**_cons**	0.04	0.00	-106.6****	0.04	0.05

Chi-square = 1034.11***

Exhibit A55: Relationships among VDC receipt and changes in hospital use over time, with sample restricted to those alive at the end of the study period (Population-averaged Model, matched sample at active sites; N = 696,336 person-months)

Variables	OR	SE	Z	95% CI	
VD	0.85	0.06	-2.37**	0.75	0.97
**month**					
**2**	1.01	0.05	0.26	0.92	1.11
**3**	1.03	0.05	0.56	0.93	1.13
**4**	1.15	0.05	2.94***	1.05	1.26
**5**	1.13	0.05	2.64***	1.03	1.25
**6**	1.11	0.05	2.19**	1.01	1.22
**7**	1.17	0.06	3.3***	1.07	1.28
**8**	1.30	0.06	5.65****	1.19	1.42
**9**	1.39	0.06	7.14****	1.27	1.52
**10**	1.53	0.07	9.49****	1.40	1.68
**11**	1.61	0.07	10.58****	1.47	1.75
**12**	1.70	0.08	12****	1.56	1.86
**13**	0.87	0.04	-2.82***	0.78	0.96
**14**	1.00	0.05	0.01	0.91	1.10
**15**	1.13	0.05	2.52**	1.03	1.24
**16**	1.10	0.05	2.06**	1.01	1.21
**17**	1.05	0.05	1.01	0.95	1.16
**18**	1.09	0.05	1.8*	0.99	1.20
**19**	1.09	0.05	1.78*	0.99	1.20
**20**	1.15	0.05	2.85***	1.04	1.26
**21**	1.06	0.05	1.29	0.97	1.17
**22**	1.12	0.05	2.32**	1.02	1.23
**23**	1.02	0.05	0.39	0.93	1.12
**24**	1.13	0.05	2.6***	1.03	1.24
**_cons**	0.04	0.00	-82.08****	0.04	0.05
Chi-square = 1034.11***					

Exhibit A56: Relationships among VDC receipt and changes in ambulatory care sensitive - hospital use over time, with sample restricted to those alive at the end of the study period (Fixed Effects Logit Model, matched among entire sample; N = 696,336 person-months)

Variable	OR	SE	Z	95% CI	
VD	1.12	0.22	0.59	0.76	1.66
NO_OFFER_POST	1.25	0.07	4.02****	1.12	1.40
**month**					
**2**	0.99	0.11	-0.12	0.80	1.22
**3**	1.11	0.12	1.01	0.90	1.37
**4**	1.17	0.12	1.46	0.95	1.43
**5**	1.31	0.13	2.67***	1.07	1.60
**6**	1.19	0.12	1.65*	0.97	1.46
**7**	1.34	0.14	2.89***	1.10	1.64
**8**	1.27	0.13	2.32**	1.04	1.55
**9**	1.43	0.14	3.52****	1.17	1.74
**10**	1.72	0.17	5.55****	1.42	2.08
**11**	1.78	0.17	5.96****	1.47	2.16
**12**	2.16	0.20	8.16****	1.79	2.60
**13**	0.91	0.10	-0.84	0.73	1.13
**14**	1.09	0.12	0.78	0.88	1.34
**15**	1.32	0.14	2.66***	1.08	1.61
**16**	1.26	0.13	2.23**	1.03	1.55
**17**	1.21	0.13	1.83*	0.99	1.49
**18**	1.02	0.11	0.22	0.83	1.27
**19**	1.19	0.13	1.67*	0.97	1.46
**20**	1.14	0.12	1.21	0.92	1.40
**21**	1.16	0.12	1.43	0.95	1.43
**22**	1.09	0.12	0.77	0.88	1.34
**23**	1.08	0.12	0.72	0.88	1.33
**24**	1.31	0.14	2.62***	1.07	1.61
Chi-square = 1034.11****					
